# The Protective Role of Symmetric Stem Cell Division on the Accumulation of Heritable Damage

**DOI:** 10.1371/journal.pcbi.1003802

**Published:** 2014-08-14

**Authors:** Peter T. McHale, Arthur D. Lander

**Affiliations:** Center for Complex Biological Systems & Department of Cell and Developmental Biology, University of California Irvine, Irvine, California, United States of America; ETH Zurich, Switzerland

## Abstract

Stem cell divisions are either asymmetric—in which one daughter cell remains a stem cell and one does not—or symmetric, in which both daughter cells adopt the same fate, either stem or non-stem. Recent studies show that in many tissues operating under homeostatic conditions stem cell division patterns are strongly biased toward the symmetric outcome, raising the question of whether symmetry confers some benefit. Here, we show that symmetry, via extinction of damaged stem-cell clones, reduces the lifetime risk of accumulating phenotypically silent heritable damage (mutations or aberrant epigenetic changes) in individual stem cells. This effect is greatest in rapidly cycling tissues subject to accelerating rates of damage accumulation over time, a scenario that describes the progression of many cancers. A decrease in the rate of cellular damage accumulation may be an important factor favoring symmetric patterns of stem cell division.

## Author Summary

Recently, highly symmetric patterns of stem cell division have been observed in a variety of adult mammalian somatic tissues. Here we identify conditions under which this behavior serves as a strategy to protect the organism against mutation accumulation. First, we find that a sufficient number of lifetime stem cell divisions must occur, potentially explaining why stem cell pools with the most symmetric divisions are rapidly cycling. Second, we find that late-occurring mutations must occur rapidly, a scenario known in cancer biology as genetic instability. These findings provide a potential explanation for the observation that cancer risks among large, long-lived organisms fail to rise as expected with lifespan and body size.

## Introduction

The accumulation of heritable damage—both mutation and epigenetic change—within individual cells is thought to be a major driver of cancer [1], [2] and aging [3], [4]. Cells employ various strategies for preventing or delaying damage accumulation including DNA repair, apoptosis and senescence [5]. Unfortunately, these strategies fail when damage lacks an immediate phenotypic consequence.

One way to delay damage accumulation in the absence of phenotypic consequences is to employ a lineage hierarchy in which self-renewing stem cells produce “transit-amplifying” cells that proliferate before differentiating into cells that eventually leave the tissue [6]. The success of this strategy relies upon transit amplifying cells being short-lived (so that damage that occurs at this stage is flushed away before more damage occurs) and stem cells dividing infrequently. However recent studies of several major vertebrate tissues (e.g. epidermis, intestinal epithelium, testis) challenge both the existence of obligatorily short-lived transit amplifying cells, and the view that stem cells usually cycle slowly [7]–[11].

**Figure 1:**
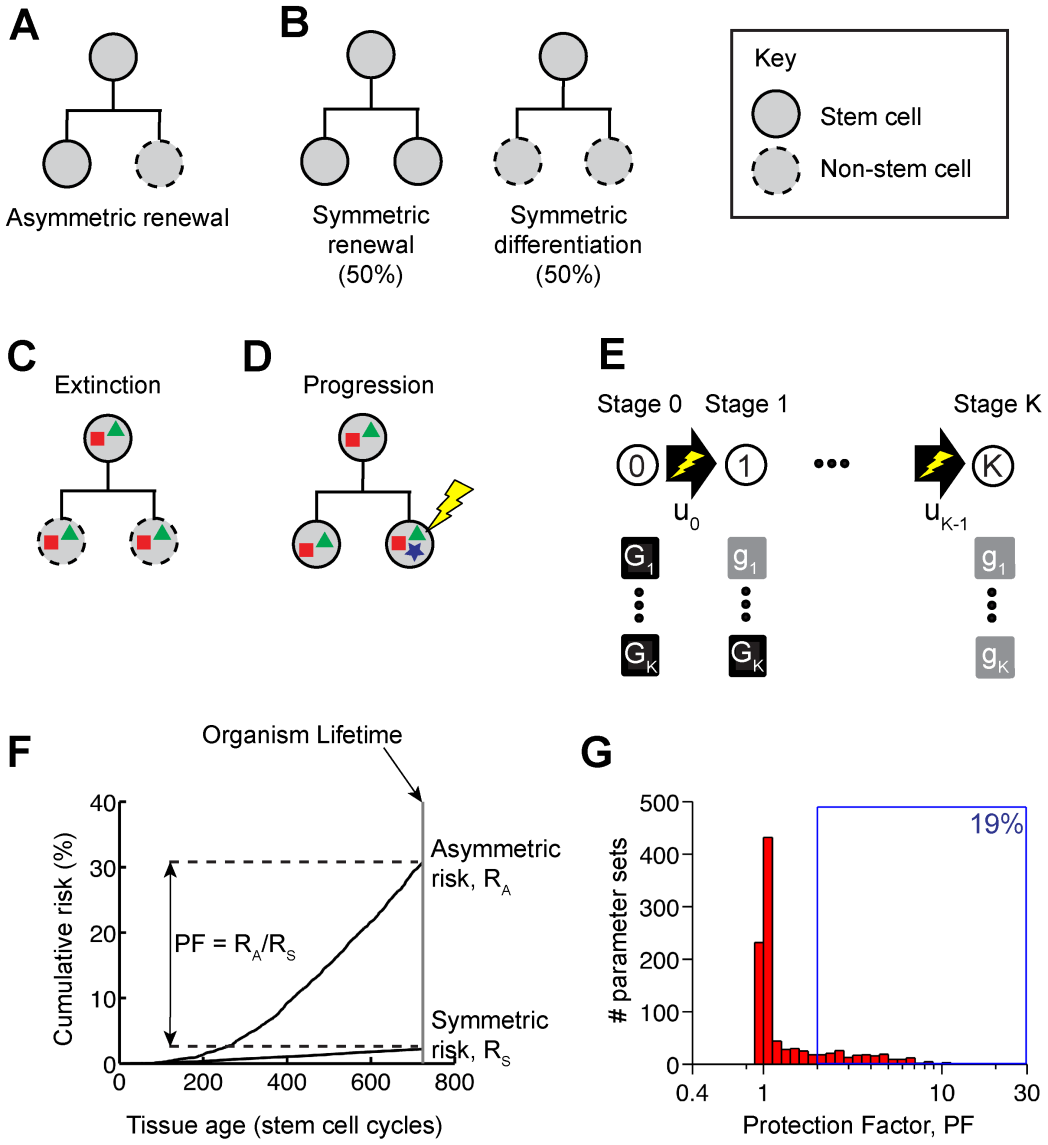
Symmetric stem cell divisions delay mutation accumulation. (A) Homeostasis of stem cell number may be achieved by invariant asymmetric renewals. (B) Alternatively, homeostasis may be achieved by balancing symmetric renewals with symmetric differentiations. (C) Stem cells and their accumulated mutations (colored polygons) are flushed out of the stem-cell compartment (“extinguished”). (D) Symmetric renewals expand the target for subsequent mutations. (E) Neutral mutations occur sequentially at loci *G_1_* through *G_K_* (Section 1.1 of Text S1). (F) A purely symmetric pattern of division reduces the risk that a population of ∼60,000 stem cells contains at least one 3-fold mutant. The mutation rates were *u_0_*∼5.10^−8^, *u_1_*∼10^−3^, *u_2_*∼5.10^−3^. (G) Symmetry reduces the probability of mutation accumulation (PF> = 1) for all parameter sets we considered (Materials and Methods; Table S1), with 19% of them exhibiting significant protection (PF>2).

The “immortal strand” mechanism [6], another proposed strategy for limiting damage accumulation, is predicated on the hypothesis that stem cells divide asymmetrically (Fig. 1A), segregating parental (less damaged) DNA strands to the daughter that remains a stem cell. Not only do recent observations question whether such DNA sorting occurs, e.g. [12], but, in vertebrates at least, most adult stem cell pools—including those of the hematopoietic system, intestinal epithelium, interfollicular epidermis, testis and hippocampus—exhibit a substantial proportion of symmetric divisions [7]–[11], [13]–[18]; (Fig. 1B).

The observation that many stem cell pools undergo symmetric divisions is interesting, given that tissue homeostasis (constancy of stem and differentiated cell numbers) demands that symmetric renewal events (where one stem cell generates two stem cells) be balanced, on average, by an equal number of symmetric differentiation events (where one stem cell generates two differentiated cells; also referred to as symmetric extinction events, since such events extinguish a stem lineage). Feedback signals from differentiated cells most likely provide such a matching mechanism [14], [19]–[21].

We were intrigued by the fact that the somatic tissue with the highest degree of symmetric stem cell division observed to date (close to 100%) is the vertebrate intestinal epithelium [10], [11], [16], because its large, rapidly dividing stem cell pool [12] ought to be particularly susceptible to mutation accumulation. Indeed, measurements of microsatellite alterations in mismatch-repair deficient mice [22], and genome-wide sequencing studies of cancer genomes (summarized in Fig. 3 of [23]), both show that the mutation burden in the vertebrate intestine is significantly higher than in other tissues. This made us wonder whether a highly symmetric pattern of stem cell division might play a role in slowing the accumulation of heritable damage. Below, we show mathematically that this is indeed the case and that, in certain biologically relevant scenarios, the protection achieved can be surprisingly large.

## Results

### Symmetric stem cell divisions delay mutation accumulation

When a stem cell undergoes an extinguishing division, it and all of its mutations (here we will use “mutation” to stand for all forms of heritable damage, genetic or otherwise) become fated to leave the body, suggesting that some of the mutation “flushing” enjoyed by short-lived transit-amplifying cells also accrues to symmetrically-dividing stem cells (Fig. 1C). Symmetric renewal divisions oppose this effect, increasing the proportion of stem cells with any given set of mutations, and elevating the risk of mutation accumulation (Fig. 1D). Since symmetric renewal and extinction must balance in homeostatic tissues (Fig. 1B), one might expect these two effects to cancel.

To test this prediction, we performed stochastic simulations of homeostatic stem cell populations of various sizes, engaging in either purely asymmetric or purely symmetric division. We allowed mutations at different loci to occur at different rates, and measured the number of stem cell divisions required for at least one stem cell to accumulate a particular number of mutations. In each simulation, the order in which specific loci mutated was fixed, allowing us to model the accumulation of mutations at *K* loci as a stepwise transition of cells through *K* stages (Fig. 1E). Later, we calculate the behavior when mutation order is not fixed (i.e. where any locus can mutate at any time).

For any cell population that chooses division outcomes stochastically, even if probabilities of renewal and extinction exactly balance, cell numbers will fluctuate around a mean value [9]–[11]; The more symmetric the division pattern, the greater the fluctuations. Such fluctuations are negligible (in relative terms) in large stem cell pools but physiologically significant in smaller ones, potentially extinguishing the entire pool. Therefore moderately sized stem cell pools that exhibit a high degree of division symmetry *in vivo*, yet don't display large size variation—for example intestinal crypts [10], [11]—must employ a variance-reducing process (e.g. size-dependent feedback control). Since the details of such processes are generally unknown, we cannot model them explicitly. Rather, we model the behavior of stem cell pools of <10^4^ cells as a Moran process (a stochastic process in which constant population size is enforced at every time point), whereas for larger pools we simulated a simple branching process without imposed size constraints (Materials and Methods). Importantly, results obtained with both approaches agreed when assessed at large population sizes (Fig. S1A).

Simulation parameters consisted of *N*, the population size (number of stem cells); *K*, the number of loci in which mutations of interest may accumulate; *u_0_*,…,*u_K-1_*, the mutation rates for acquiring each of the *K* mutations; and *L*, the organism lifetime measured in stem cell cycles. For each set of parameters we determined the fraction of simulations of purely asymmetric division in which at least one stem cell had *K* mutations—the “asymmetric risk”—as a function of time (see Materials and Methods). The “symmetric risk” was calculated similarly, but employing a purely symmetric division pattern. One possible way to quantify the difference between the two risks (at otherwise identical parameter values) is to measure displacement, along the time axis, from one risk curve to the other, i.e. the amount of extra time a particular division strategy confers on a stem cell pool before it acquires a cell with *K* mutations. Though such a “mean first passage time” approach is mathematically sound, the answer one obtains is biologically irrelevant whenever the mean-first passage time is much shorter or much longer than the reproductive lifespan of the organism. We therefore measured the ratio of risks at a single time point, which we term the “Protection Factor” (PF), because a change in the probability of having a deleterious phenotype (*K* mutations in at least one stem cell) at a fixed time point (e.g. the end of an organism's reproductive period) is directly connected to the pressures of natural selection at the organism level.

Care must be exercised in choosing the time at which PF is evaluated since, with enough time, all risks plateau at 100%. Accordingly, PFs were typically ascertained when the asymmetric risk (always greater than or equal to the symmetric risk; see below) lay in the vicinity of 50% (Materials and Methods), i.e. at a time when a stem cell pool executing only asymmetric divisions would have a 50% chance of possessing at least one *K*-fold mutant stem cell. Later, we also consider cases of many small stem cell pools functioning in parallel (i.e., a compartmentalized population), for which much smaller asymmetric risks must be used.

Fig. 1F presents the results for a particular case in which the size of the stem cell pool was *N*∼60,000 stem cells and the number of accumulated mutations was *K* = 3. The symmetrically dividing stem cell population clearly displays a significantly reduced risk of arriving at the fully mutant state. Fig. 1G summarizes the results for 1000 parameter sets in which population sizes, mutation rates, and number of mutable loci (*K*) were chosen at random, subject to the condition that the time (in cell cycles) when the asymmetric risk was close to 50% should fall within a reasonable range of organism lifetimes (Table S1). Interestingly, 19% of simulations exhibited PF>2, i.e. symmetric division cut the risk of mutation accumulation by at least half. This suggests that significant protection against mutation accumulation occurs over a substantial fraction of parameter space.

### Highly protected parameter sets exhibit characteristic dynamics

**Figure 2:**
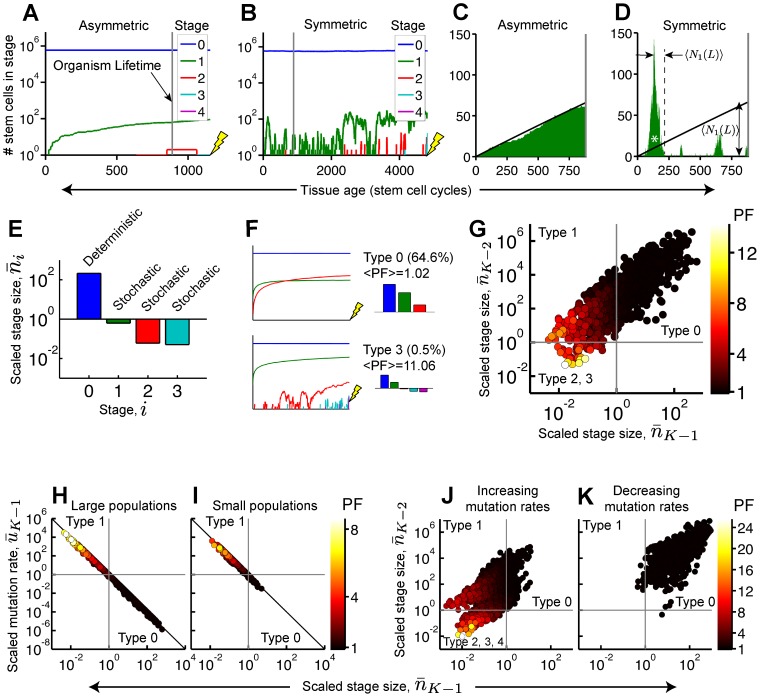
Characteristic dynamics of highly protected parameter sets. (A, B) Typical dynamics of a highly protected parameter set contrast how the first 4-fold mutant stem cell (yellow lightning bolt) is acquired in asymmetric (A) versus symmetric (B) populations. Vertical grey line indicates organism lifetime. Mutation rates are *u_0_*∼10^−7^, *u_1_*∼10^−4^, *u_2_*∼5.10^−3^, *u_3_*∼5.10^−3^. (C, D) The number of stage-1 stem cells closely tracks its mean value (black line; Eq. (S5)) in the asymmetric population (C), but frequently fluctuates down to zero in the symmetric case (D). (E) Scaled stage sizes for the parameter set analyzed in (A–D). (F) Parameter sets were classified based on the number of stochastic stages (Fig. S1B). Symmetric trajectories representing two of the classifications are shown. <PF> is PF averaged over all parameter sets in a particular class. (G) Parameter sets containing many strongly stochastic stages (bottom-left) are highly protected (hot colors). (H, I) Simulated data (circles) collapse onto the scaling curve (line) defined in Eq. (2) in both large (H) and small (I; Section 2.1 of Text S1; Table S2) populations. Type-2 and -3 parameter sets (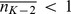; see panel F) were removed from the analysis. (J, K) Numerical screen (Table S3) subject to the constraint that mutation rates are monotonically “increasing” (J) or “decreasing” (K). Most (52.7%) of the parameter sets are significantly protected (PF>2) in the “increasing” case compared with none in the “decreasing” case.

To understand the origin of protection, we examined the dynamics of mutant stages (i.e. sub-populations with a given number of mutations per stem cell) in individual simulations. Stage size fluctuated more when divisions were symmetric versus asymmetric (Fig. 2A, B); this is expected because, in symmetrically-dividing populations, fluctuations come not only from the random entry and exit of cells as they acquire mutations, but also from transient imbalances in renewal versus extinction.

Fluctuations in one stage are expected to alter the rate of entry of cells into the next stage, depending upon the size and direction of the fluctuation. As long as upward and downward fluctuations balance—which they usually do—they should have no long-term effect on rates of mutation accumulation, and therefore offer no protection. There is one circumstance under which they will not balance, however, which is when fluctuations extinguish all the mutants in a given stage. In that case that stage must await the entry of a new mutant cell from the previous stage before upward fluctuations can resume; in principle, this effect could slow the rate of mutation accumulation. Consistent with this idea, when we visually examined simulations of symmetrically dividing populations that exhibited high PF, we always observed frequent extinctions of one or more mutant stages (Fig. 2D).

If stage extinctions are the basis for protection, then the magnitude of protection might reflect the propensity of stages to extinguish before they progress (i.e. acquire a subsequent mutation). In other words, for protected cases we expect the average time for a stage to extinguish to be much smaller than the average time to progress. For stages 1, 2 and 3 in Fig. 2B, we see that progression occurs only once a rare clone expands to a large size. The largest stage-*i* clone that arises during an organism lifetime (e.g. the clone indicated by an asterisk in Fig. 2D) extinguishes in a time of order 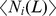, where 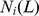 is the (random) number of stage-*i* stem cells at the end of life, *L*, and 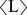 represents an average over many realizations of the stochastic process (derived in Section 1.5 of Text S1). On the other hand, were that clone dividing asymmetrically, the mean time to acquire a new mutation would have been 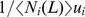. Thus, clonal extinctions outcompete progression when 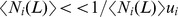, which can be conveniently formulated as 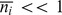
_,_ where 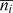, the “scaled stage size,” is defined by
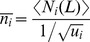
(1)Stages with 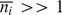 exhibit time-dependent trajectories that are well described by the “deterministic” equations, Eq. (S5), whereas stages with 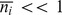 are “stochastic” (Fig. 2E). Parameter sets with multiple stochastic stages are, on average, the most highly protected (Fig. 2F). In Fig. 2G we correlate the observed scaled stage size for the penultimate and antepenultimate stages (i.e. stages *K-1* and *K-2*), color-coding the data by the observed PF; significant protection only occurs when 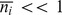 for the penultimate stage, and is largest when 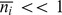 for both stages. In general, protection increases with the number of consecutive stages satisfying 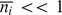 (Fig. S1B).

What sorts of parameter values give rise to such behavior? We established a simple scaling relation (Section 1.5.2 of Text S1 and illustrated in Fig. 2H, I)
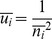
(2)for the “scaled mutation rate” defined by
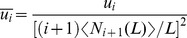
(3)
Eq. (2), implies that when a stage is protected (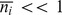), the scaled mutation rate is necessarily large, 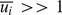. The latter condition is less stringent at later stages, where the threshold mutation rate 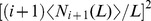 from Eq. (3) is typically smaller, suggesting that protection is favored by high mutation rates at late stages. Indeed, numerical screens, conducted subject to the constraint that mutation rates are strictly accelerated (versus decelerated) after an arbitrarily chosen stage, show dramatic enrichment of protected parameter sets (Fig. 2 J, K).

### Rapidly renewing tissues are most protected

**Figure 3:**
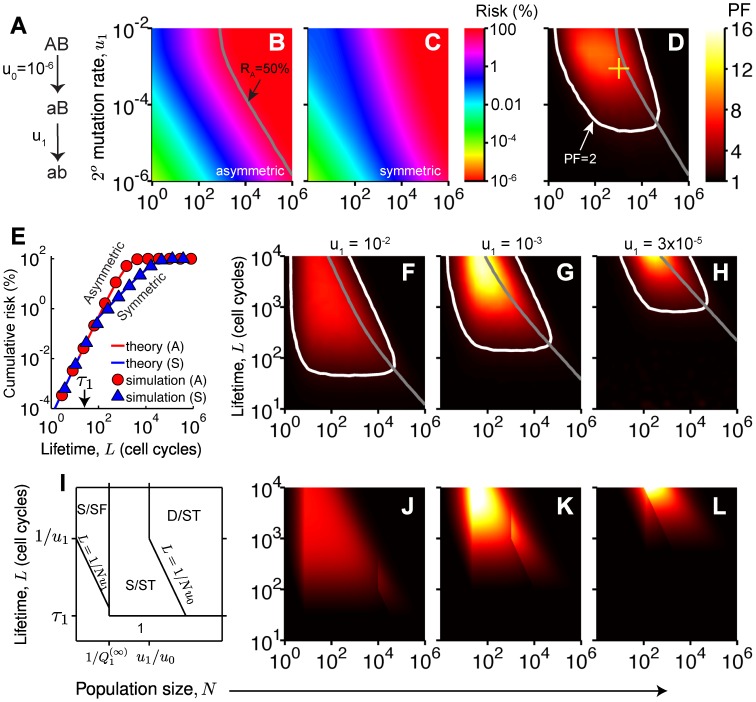
Rapidly renewing tissues are most protected. (A) Accumulation of two mutations. (B, C) Cumulative risk of generating at least one double mutant in asymmetric (B) versus symmetric (C) populations (organism lifetime is 10^3^ stem cell cycles). Heat maps have been linearly interpolated. *R_A_*, asymmetric risk. (D) PF heat map obtained by dividing panel B by C. (E) Cumulative risk as calculated using Monte Carlo simulation (symbols) and Eqs. (S48, S55, S56) (lines). Population size is *N* = 10^3^ stem cells and mutation rates are *u_0_* = 10^−6^ and *u_1_* = 10^−3^ (yellow cross in (D)). (F–H) PF heat maps for a spectrum of secondary mutation rates, *u_1_*. (I) Regimes where PF formulae are valid (Section 2.3.1 in Text S1). Asymmetric risk is approximated by Eqs. (S48) and (S54), represented here by S and D, respectively whereas symmetric cumulative risk is approximated by Eqs. (S55) and (S59), represented by ST and SF, respectively. The transitions between regimes are not sharp but represent smooth crossovers. (J–L) PF calculated using the piecewise formula (see (I)) for the same parameter values as (F–H). The formula is accurate to 40% throughout the protected zone (PF>2) (Fig. S2P–R).

To gain further insight, we focused on cases of mutation accumulation with just two mutant stages (Fig. 3A). With large stem-cell populations, mutation rates must be unrealistically slow to meet the requirement that asymmetric risk at biologically realistic lifetimes should be ∼50%; we therefore simulated pools of 1 to 10^6^ stem cells (using a Moran model). Fig. 3B and C show the asymmetric and symmetric risks as a function of the secondary mutation rate and population size, with the lifetime and primary mutation rates held fixed (at 10^3^ cell cycles, and 10^−6^ per cell cycle, respectively). The grey contour marks parameter combinations for which the asymmetric risk is 50% at the organism lifetime (i.e. the conditions under which risks were compared in Fig. 2). Panel D, which plots the ratio of panels B and C, i.e. PF, shows that significant protection can occur in a large region of parameter space (circumscribed by a white contour line), with protection as great as 16-fold possible. As concluded in the previous section, a high final mutation rate (i.e. *u*_1_≫*u*_0_) is necessary to achieve significant protection.

To uncover the role of the tissue renewal rate, we varied the number of stem cell cycles that occur during the organism's lifetime. Fig. 3E indicates that, though protection is impressive (PF>2) in rapidly cycling tissues (i.e. those tissues whose stem cells cycle at least 100 times during the organism's lifetime), it vanishes (PF = 1) in slowly cycling tissues (small number of cell cycles). This dependence of protection on lifetime stem cell output, which we would have missed had we simply gauged protection from mean first passage times (e.g. the times in Fig. 3E at which each risk reaches 50%), is also seen for other population sizes and secondary mutation rates (Fig. 3F–H). We derived a piecewise analytical formula for PF (Section 2.3 in Text S1; Fig. 3I) that is an excellent approximation of the simulated results, as seen by the excellent fitting of symbols in Fig. 3E, and by the resemblance of the simulation heat maps in Fig. 3F–H with their analytical approximations in Fig. 3J–L. This analysis shows that significant protection is expected when the organism lifetime is appreciably larger than the mean time it takes a symmetrically dividing, single-mutant clone to progress to the next stage. In larger populations, where the clone is unlikely to fix (Fig. S2D; “Stochastic Tunneling” regime), this progression time is
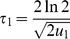
(4)(Fig. 3E, I; Section 1.7 of Text S1) whereas it is 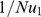 in smaller populations (Fig. 3I), in which the clone first fixes before progressing (Fig. S2L; “Sequential Fixation” regime). In both regimes, protection is favored by minimizing the clonal progression time, which, these formulae tell us, occurs when the secondary mutation rate is fast, as concluded in the previous section (Fig. 2). In short, the observations that rapid stem cell divisions *or* fast terminal mutation rates favor protection are in fact just two sides of the same coin (Section 2.3.1 of Text S1).

### Even modest amounts of symmetry provide significant protection

**Figure 4:**
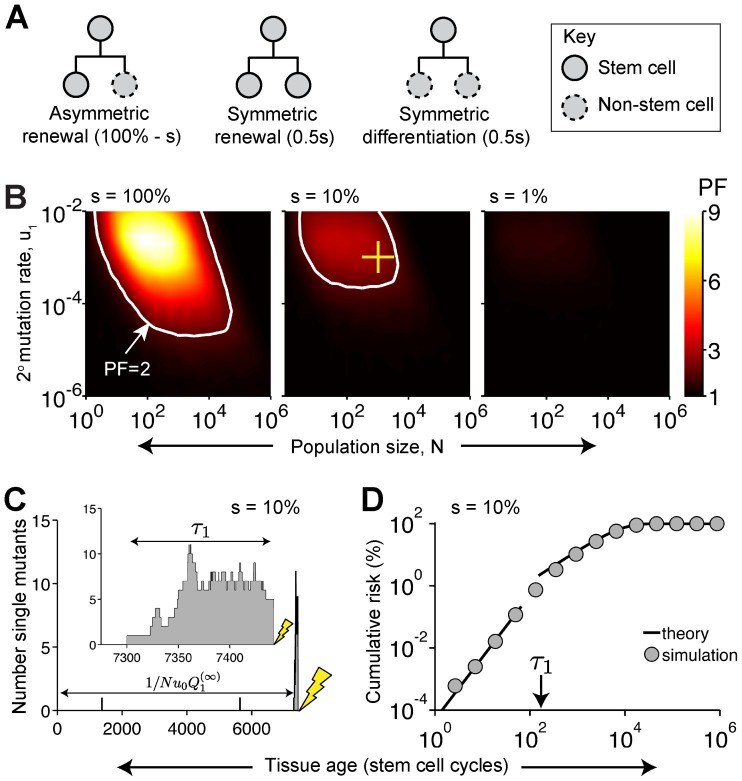
Modest amounts of symmetry can provide significant protection. (A) A fraction *s* of stem cells divide symmetrically while the others divide asymmetrically. (B) Protection against the accumulation of two mutations. Lifetime is *L* = 10^3^ stem cell cycles and mutation rate is *u_0_* = 10^−6^. (C) Typical dynamics, with blow-up of the last few cell cycles (inset). Horizontal arrows indicate the mean time that a single-mutant lineage drifts before mutating, Eq. (S30), and the mean time until the production of the first single-mutant clone destined to mutate, 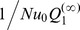 (c.f. Eq. (5)). *N* = 10^3^ stem cells and mutation rates are *u_0_* = 10^−6^ and *u_1_* = 10^−3^ (cross in panel B). (D) Corresponding cumulative risk from simulation (symbols) and Eqs. (S55) and (S56) (lines).

So far, we have assessed the protection offered by a purely symmetric pattern of stem cell renewal, yet many tissues (e.g. the mammalian epidermis [7], [8]) employ a mixture of asymmetric and symmetric stem cell divisions (Fig. 4A). How much should “contaminating” asymmetric divisions reduce protection? Surprisingly, we find that a tissue employing symmetric divisions just 10% of the time still robustly delays mutation accumulation (Fig. 4B) via extinctions of intermediate-stage clones (Fig. 4C), just as we found in purely symmetric cases (e.g. Fig. 2B). A formula for the probability that a single-mutant clone mutates (derived in Section 1.6 of Text S1 and used to plot the theoretical curves shown in Fig. 4D)
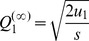
(5)(*s* is the fraction of divisions that are symmetric) explains why: new clones likely extinguish so long as *u_1_≪s*, i.e. provided the mutation rate is slow compared to the symmetric-division rate—not a particularly stringent condition, even with an elevated mutation rate.

### Order of mutation is critical

**Figure 5:**
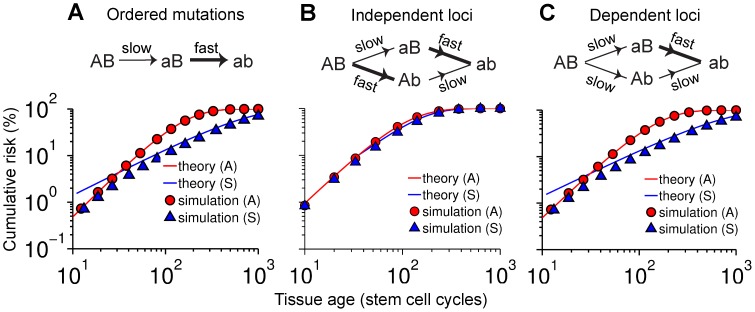
Mutation order is critical. (A) Locus A always mutates before locus B. Theoretical asymmetric risk (red) is Eq. (S11) with *K* = 2, 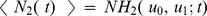, and *H_2_* defined in Eq. (S49). Theoretical symmetric risk (blue) is Eq. (S55). (B) Either locus may mutate first. Reversing the order in which the loci are mutated reverses the order of the mutation rates since the loci are independent. Theoretical predictions are Eqs. (S66) (red) and (S68) (blue). (C) If the mutation rate of a locus depends on the genomic background then all four mutation rates may be independently chosen. Here, the rate at which B mutates depends on whether A is mutated or not. Theoretical predictions are the same as panel A. In all panels, population size is 10^4^ stem cells and mutation rates are 10^−6^ (“slow”) and 10^−2^ (“fast”). Panels A and C show the results of simulations under the “Moran” model (Section 3.2 of Text S1) whereas panel B corresponds to the “Branching” model (Section 3.1).

We saw that symmetric stem cell divisions are protective when fast mutations occur late (Fig. 5A; Fig. 2J) but not when they occur early (Fig. 2K). If mutations are independent, however, fast mutations sometimes occur early and sometimes late (Sections 3.1 and 3.2 of Text S1). Under such circumstances, little if any protection is observed (Fig. 5B; Fig. S3D–H) because most double mutants arise via the fast-slow route (which is not protected), rather than the slow-fast one (which is protected).

One important scenario where mutation rates are not independent is the development of cancer, where alterations at “genetic stability” loci—whether by DNA sequence changes or aberrant epigenetic alterations—elevate mutation rates throughout the genome (see Discussion). Fig. 5C presents the case where A represents a genetic stability gene, so that B mutates rapidly *only if* A is already inactivated. In this scenario, the unprotected path (genetic stability gene mutated last) can no longer compete with the protected path (genetic stability gene mutated first). Thus protection conferred by division symmetry persists. It should be noted that, for these calculations, mutations in A were treated as neutral (i.e. not by themselves affecting fitness), which is valid as long as the increase in mutation rate is small enough that the added lifetime burden of subsequent deleterious mutations is inconsequential.

### Effect of compartmentalizing stem cell populations: The mouse small intestine

**Figure 6:**
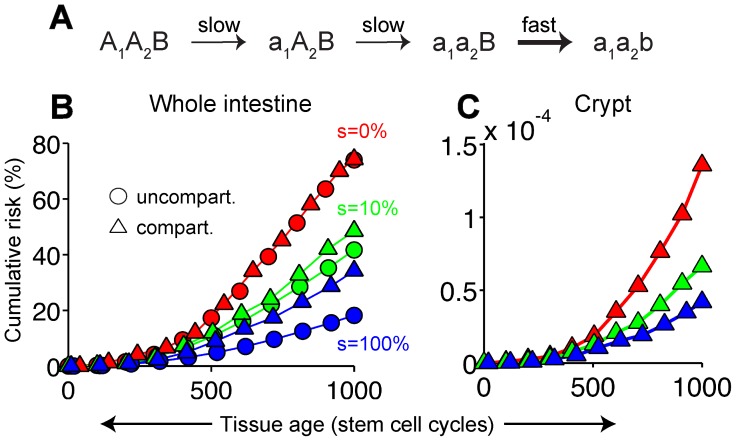
Compartmentalization of the mouse intestine. (A) Slow inactivation of both copies of a genetic stability gene (loci A_1_ and A_2_) followed by rapid mutation of another gene, B, as a result of genetic instability. (B, C) The corresponding cumulative risk of generating at least one triple-mutant stem cell in the whole intestine (B, circles) and in an individual crypt (C), as calculated using Monte Carlo simulations. In the whole-intestine case, cumulative risk was re-computed assuming independent crypts (B, triangles). Lines are guides to the eye only. Mutant clones progress to the next stage via mechanisms known to population geneticists as “Stochastic Tunneling” in the un-compartmentalized case (Fig. S4B) and “Sequential Fixations” in the compartmentalized case (Fig. S4C). Mutation rates are 10^−6^ (‘slow’) and 10^−3^ (‘fast’). The mouse small intestine was assumed to contain ∼10^6^ crypts [24] each containing ∼10 Lgr5+ stem cells dividing ∼10^3^ times during the mouse' lifetime [25].

Mutation accumulation is expected to be particularly acute in the mouse small intestine because its calculated lifetime proliferative output—some 10^10^ stem cell divisions [24], [25]—is extraordinarily high. Under a plausible mutation progression scenario (neutral mutations in a genetic stability gene followed by rapid mutation of APC [26]–[28]; Fig. 6A) simulations show that as little as 10% symmetric divisions are significantly protective (circles in Fig. 6B). This calculation assumes, however, that mutant clones expand unimpeded whereas, in reality, stem cells are segregated into crypts, with clonal expansion beyond crypt boundaries occurring only infrequently (1–10 times per crypt per lifetime [29], [30]). We therefore accounted for this fact by computing lifetime risk in an individual crypt (Fig. 6C) and then integrating that risk over all crypts in the intestine. Fig. 6B (triangles) shows that compartmentalizing in this way does not affect the intestine-wide asymmetric risk (as expected since, in that scenario, each stem cell behaves independently), but does increase the symmetric risk, although it is still substantially lower than the asymmetric risk.

## Discussion

We show here that observed levels of division symmetry in vertebrate tissues [7]–[11] can lower the risk of heritable damage accumulation even if the amount of symmetry is modest, the tissue is compartmentalized, or damage is initially phenotypically silent. For this effect to be physiologically significant—substantially reducing a high cumulative incidence of mutation—conditions must favor the frequent stochastic extinction of multi-hit stages (e.g. 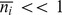; see Eq. (1)), which places significant constraints on mutation rates and the order in which mutations occur.

Even when we relax the assumption that damage is phenotypically silent (e.g. by allowing mutations to be selectively advantageous), we find that protection can persist, provided that selection coefficients are 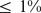 (Fig. S5; see also Section 4 of Text S1). Recent analyses of cancer genome data suggest that cancers commonly evolve through multiple driver mutations [31] with selection coefficients on the order of 1% (assuming normal mutation rates) or lower (assuming genetic instability) [32]. Thus our results should be relevant not only to the accumulation of neutral mutations but also to mutations that drive cancer evolution.

Others have noted that patterns of division symmetry should have an impact on the stochastic dynamics of cancer stem cells (e.g. how division pattern influences the probability of clonal fixation [33] or the rate at which drug resistance should develop in a growing tumor [34]) whereas here we focused on the accumulation of neutral mutations that precede the development of cancer. Although some authors have dismissed the idea that division symmetry can have any effect on neutral mutation accumulation [35], a few studies have identified specific scenarios in which symmetry can be protective [36]–[38]. None of these studies systematically identified the extent of protection as a function of mutation rate, population size, and organism lifetime. By doing so here, we discovered that necessary and sufficient conditions for protection are: (1) a large enough number of stem cell divisions must occur over the organism's lifetime (Fig. 3E–H); (2) late mutations must occur rapidly (e.g. 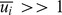; see Eq. (3); Fig. 2H, I).

The first condition arises because there need to be enough stem cell divisions for a substantial number of lineage extinctions to occur. This is likely the case in many mammalian tissues (e.g. epidermis, intestine, testis [7], [9], [11]), but probably not in the somatic tissues of small, short-lived animals. This difference may help explain why most observations of purely (or predominantly) asymmetric stem cell division have come from studies of invertebrates such as *Drosophila* [39]–[42]—in which the protective effect of symmetry is likely to be virtually nil—whereas most observations of substantial symmetry come from studies of mouse, cat, monkey and man [7], [9]–[11], [13], [15], [17], [43].

The second condition for significant protection—that late mutational steps be fast (Fig. 2J)—broadens the distribution of latencies to arrive at the final mutant state (Fig. S6). This is an example of a general principle: distributions formed by sequential stochastic processes become over-dispersed when late steps occur on a faster time scale than earlier ones. Another example is the supra-Poissonian variance in protein levels seen among cells when mRNAs are translated more rapidly than they are transcribed [44].

That protection requires late mutations to be rapid suggests that the primary physiological value of symmetric stem cell division is cancer-risk reduction. This is because genetic instability is thought to play a key role in the development and progression of many cancers, especially at late stages [45]. Mutations in genes associated with DNA polymerase proofreading [46]; DNA damage repair [47], [48]; chromosome segregation [49], [50]; chromatid cohesion [51]; DNA damage checkpoints [52]; as well as aberrant epigenetic alterations [53]; appear commonly in human cancers and/or the germline of individuals pre-disposed to cancer. The effect of these genetic or epigenetic alterations is to make other loci mutate more rapidly, creating just the scenario in which division symmetry will be protective (e.g. Fig. 5C).

Levels of genetic instability in cancer can be large. Studies of human tumors and their adjacent normal tissue find a mutational load of 0.1–100 somatic variations per megabase, a significant fraction of which exhibit mutagen signatures, e.g. [54], [55]. Colorectal tumor samples harboring alterations in more than one genetic stability gene contain 10–100 variants per megabase [47], which is 10- to 100-fold more than expected given a normal mutation rate [56]. Not only is mutation prevalence increased in cancer, so are mutation rates. When compared with normal cells, human colorectal carcinoma cell lines with DNA mismatch repair loss exhibit a 100- to 1000-fold increase in mutation rate [57], [58], and even those without this deficiency display a 10- to 100-fold increase in loss or gain of entire chromosomes [59].

Is this amount of genetic instability high enough to make protection physiologically significant? With a 1000-fold acceleration in mutation rate, fully symmetric division could lower mutation accumulation risk in the mouse intestine by 2.2-fold (Fig. 6B). Extrapolating to stem cell numbers and lifetimes representative of the human intestine, we expect protection to increase a *further* 2.5- to 3.6-fold at ages 30 and 60, respectively (Fig. S7A, B; see also Section 5 of Text S1); indeed even with only a 100-fold acceleration in mutation rate, calculations indicate that symmetric stem cell divisions still provide significant protection (PF = 1.5 and 1.9 at ages 30 and 60, respectively; Fig. S7C, D).

A direct test of the hypothesis that symmetric stem cell division lowers cancer risk would require experimental manipulation of division patterns. The molecular mechanisms that produce highly symmetric assignment of cell fates are unknown, although it should be noted that symmetry fractions between 50% and 100% arise spontaneously if cells simply chose their fates at random (a 50% level is achieved if fate is exclusively determined after division; a 100% level if fate is exclusively determined before). Accordingly, the acquisition of highly symmetric division patterns may be less about implementing special mechanisms than about not implementing mechanisms required to guarantee asymmetric divisions.

Although we have evaluated the effect of mutation accumulation in a single cell type, our results are easily generalized to multi-stage lineages, in which intermediate cell stages (“committed progenitors”) may be modeled as products of stem cells whose rates of differentiation exceed those of renewal (allowing for a steady-state influx of earlier-stage cells). This imbalance by itself enhances the flushing of mutants arising at such stages. Any further propensity for symmetric divisions beyond this minimum level would provide further protection against mutation accumulation, in precisely the same way as described above for stem cells.

Recently, Roche et al. argued that the documented lack of an expected inter-species correlation between cancer risk and body size/longevity (“Peto's paradox”) implies that large, long-lived species must have evolved strategies to reduce cancer risk [60]. Here, we identify symmetric stem cell division as one such strategy. Whether the protection this strategy offers is sufficient to explain natural selection for symmetry in the ancestors of mammals is difficult to know, especially since we do not know the conditions under which selection took place. It may be enlightening to determine whether a prevalence of symmetric vs. asymmetric division patterns coincides with the emergence of larger, longer-lived species.

## Materials and Methods

### Mathematical models

We modeled mutation accumulation in large populations of stem cells with a discrete-time branching process where each division produces 0, 1 or 2 stem cell daughters, each of which randomly accumulates a mutation (Section 1.1 and 1.2 of Text S1). Small populations were treated using a continuous-time Moran-type model (Section 2.1 of Text S1). Populations, initially mutation-free, were simulated until either a *K*-fold mutant stem cell appeared or the simulation reached the organism's lifetime. For each parameter set, we generated ∼10^3^ *successful* runs in which the mutant appeared within a lifetime, which was sufficient to estimate the lifetime cumulative risk of mutation accumulation to an accuracy of ∼3% (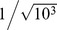). For example, approximately 2000 *total* runs were required when the risk was ∼50% (e.g. Figs. 1, 2) but as many as 10^11^ *total* runs were needed for risks of the order of 10^−6^% (risk per human crypt in Fig. S7). For computational reasons, we updated our risk estimates after each run.

### Numerical screen

The number of accumulated mutations was sampled from a uniform distribution typically defined on the interval 1 to 10 whereas log-parameter values of population size, organism lifetime and mutation rates were sampled from a uniform distribution on the log of the ranges (Tables S1, S2, S3). We repeatedly generated parameter sets until we obtained 1,000 parameter sets in which the predicted asymmetric lifetime risk R_A_ (Eq. (S12)) lay in a defined range (10%<R_A_<99.996%). For each such parameter set, we then simulated the asymmetric and symmetric risks (Fig. 1F).

## Supporting Information

Figure S1 **Numerical screen.** (A) Concordance of “Moran” and “Branching” models used to screen large and small populations, respectively. The lifetime cumulative risk of accumulating two mutations in a symmetric population was computed for a variety of stem-cell population sizes and organism lifetimes under both models (right panels). The models predict the same cumulative risk over most of parameter space but differ significantly in small populations at large lifetimes, where extinctions of the entire stem cell population in the Branching model reduce risk by at least a factor of two (white contour in left panel). (B) Parameter sets comprising the numerical screen of Table S1 were classified into 4 types based on the number of stochastic stages. Representative symmetric trajectories are shown. Notice the correlation between the number of stochastic stages and mean PF (averaged over all parameter sets with a given number of stochastic stages).
(PDF)

Figure S2
**Analysis of stochastic tunneling and sequential fixation regimes.** (A, B) A single wild-type stem cell was simulated until either one of its descendants mutated (with probability 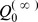) or its lineage extinguished without mutating (with probability 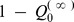). The mean time that a branching lineage drifts before mutating, 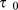, was recorded in those cases where mutation occurred. Panel A shows that the simulated lineage mutation probability (symbols) is well described by Eq. (S24) (lines) whereas panel B shows that the simulated drift time (symbols) is well described by Eq. (S30) (lines). (C–F) Typical dynamics at long times (C, D) and short times (E, F) prior to the production of the first double-mutant stem cell (yellow lightning bolt). Inset to (D) is a magnified view of the last few generations of the simulated dynamics. Population size is *N* = 10^3^ stem cells and mutation rates are *u_0_* = 10^−6^ and *u_1_* = 10^−3^. (G–I) Protection vanishes for small secondary mutation rate, *u_1_*≪1/*L* ^2^. In these panels, population size is *N* = 10^3^ stem cells and mutation rates are *u_0_* = 10^−6^ and *u_1_* = 10^−8^. (G) Simulated (symbols) and theoretical (line, Eq. (S56)) cumulative risk. (H, I) Typical trajectories that generate a double-mutant stem cell by end of life, *T_2_*<*L* = 10^3^ cc. In both cases, one of the first few single-mutant lineages to arise from the wild-type background produces a double-mutant stem cell that arises improbably early in its parent single-mutant lineage, *T_2_*≪
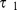
≪1/*u_1_*. (J–O) Sequential Fixation Regime. In these panels, population size is *N* = 10 stem cells and mutation rates are *u_0_* = 10^−6^, *u_1_* = 10^−4^. (J) Cumulative risk calculated using simulation (symbols) and Eqs. (S48), (S56) and (S59) (lines). (K, N) A double-mutant stem cell typically arises in the first single-mutant stem cell in a purely asymmetric population. (L, O) Dynamics in a purely symmetric population. (L) At long times, *t*≫1/*Nu_1_*, single-mutant lineages frequently extinguish before one survives drift, fixes in the population, and then rapidly acquires the next mutation. The time taken to fix, *N*, and to acquire the second mutation once fixed, 1/*Nu_1_*, are negligible compared to the time taken for a single-mutant lineage destined for fixation to arise, 1/*u_0_*. (O) At short times, *t*≪1/*Nu_1_*, the time between fixation and mutation cannot be neglected. (M) Symmetric extinctions out-compete fixation events to reduce mutation accumulation risk. (P–R) Accuracy of piecewise analytic formula for PF measured by the ratio of analytically computed PF (Fig. 3J–L) to exact PF as calculated via Monte Carlo simulation (Fig. 3F–H). Grey contours delineate regions (red) where the fractional error of the analytic formulae |PF_analytic_ - PF_exact_|/PF_exact_ is less than 40%, including practically all the protected zone (PF_exact_>2; white contour). Panels A and B were generated under the Branching model, Eq. (S1), whereas panels C–R were generated using the Moran model, Section 2.1 of Text S1. In panels C–O, purely asymmetric (symmetric) trajectories are in red (blue). cc, cell cycles.(PDF)

Figure S3
**Unordered “fast” and “slow” mutations.** (A–C) A “fast-slow” ordered pathway. (A, B) The abundance of *Ab* stem cells is approximated by its mean value, 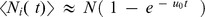, which follows from Eq. (S51) when *t*, *1/u_0_*≪1/*u_1_*. (C) Simulated cumulative risk (symbols) is approximated by 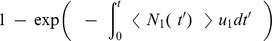. (D–H) Dynamics at short times, t≪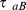, of unordered “fast” and “slow” loci. The black line in panels F and G is Eq. (S67). (H) Simulated cumulative risk (symbols) is approximated by Eq. (S71). In all panels, population size is *N* = 10^4^ stem cells and mutation rates are 10^−2^ (“fast”) and 10^−6^ (“slow”). Time courses are plotted until the first double-mutant stem cell appears in the entire stem cell population.(PDF)

Figure S4 **Clonal extinctions out-compete progression in the intestine.** (A) Model of mutation accumulation in the intestine. (B) Typical dynamics showing how various patterns of division generate the triple-mutant stem cell in the un-compartmentalized case. (C) Dynamics in a single crypt of a compartmentalized intestine. The purely asymmetric trajectory (*s* = 0%) is representative of all trajectories examined whereas the mixed (*s* = 10%) and purely symmetric (*s* = 100%) trajectories show the most frequently observed type since all four possible combinations of stochastic tunneling and sequential fixation were observed at appreciable frequencies (see also Ref [17] in Text S1). The intestine was assumed to comprise 10^6^ crypts, each containing 10 stem cells. Mutation rates are 10^−6^ (slow) and 10^−3^ (fast).
(PDF)

Figure S5 **Protection persists when selection acts on stochastic stages.** Protection against ordered accumulation of *K* = 2 mutations after 1000 stem cell cycles for symmetry fractions *s* = 100% (A) and 10% (B), calculated by Monte Carlo simulation of the generalized model presented in Section 4.1 of Text S1. The selection coefficient is defined in the model by (*w_1_-w_0_*)/*w_0_*, where *w_i_* is the fitness of stage *i* (see Section 4.1 of Text S1). The insensitivity of PF to wide variations in the selection coefficient is an example of the general principle in population genetics that selection is ineffective provided the magnitude of the selection coefficient is smaller than the inverse population size. Mutation rates are *u_0_* = 10^−6^ and *u_1_* = 10^−3^ per locus per stem cell cycle.
(PDF)

Figure S6
**“Increasing” mutation rates yield a broad distribution of latencies.** Probability distributions of times at which the first double-mutant stem cell arose in a population of *N* = 10^4^ symmetrically dividing stem cells, from simulations of the discrete-time branching process defined by Eq. (S1) (bars) and from the probability mass function 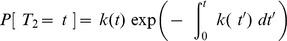 (lines). In the Deterministic regime (A), the rate constant is given by 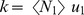, where the mean abundance of single-mutant stem cells is 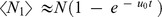, whereas it is 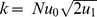 in the Stochastic Tunneling regime (B). When the mutation rates are decreasing, the distribution of latency until the first double-mutant stem cell is narrow (A), but when the mutation rates are increasing, the distribution becomes wider (B), even at the same mean latency (858 cell cycles in both cases). Histogram bar height represents the probability that the mutation occurred between the bar edges. Insets show typical stochastic realizations (red) and mean single-mutant abundance (black).(PDF)

Figure S7 **Protection in the human colon.** Cumulative risk of ordered accumulation of *K* = 4 mutations by ages 30 (A, C) and 60 (B, D), assuming mutation rates increase 1000-fold (A, B) or 100-fold (C, D) during the course of mutation accumulation from an initial rate of *u_0_* = 5×10^−7^ per locus per stem cell cycle; see Ref [18] in Text S1. Lines are Eqs. (S75) and (S76) whereas symbols are Monte Carlo simulations (under the Moran model; Section 2.1 of Text S1). The colon is assumed to be compartmentalized into *M* = 10^7^ crypts, (∼10^4^ crypts/cm^2^×∼10^3^ cm^2^/colon; Ref [19] of Text S1) each containing *N* = 20 stem cells (Ref [20], [21] of Text S1) dividing purely asymmetrically (red) or symmetrically (green) 100 times per year. Mutation rates of consecutive stages (*u_0_*; *u_1_*; *u_2_*; *u_3_*) are (A) 5×10^−7^; 5×10^−7^; 5×10^−6^; 5×10^−4^; (B) 5×10^−7^; 5×10^−7^; 5×10^−7^; 5×10^−4^; (C) 5×10^−7^; 5×10^−7^; 5×10^−5^; 5×10^−5^; (D) 5×10^−7^; 5×10^−7^; 5×10^−6^; 5×10^−5^.
(PDF)

Figure S8 **Symmetry protects even when mutations may occur simultaneously in both daughter stem cells.** The more accurate ordered mutation accumulation model presented in Section 6 of Text S1 was used to generate a distribution of PFs over a random ensemble of parameter sets equivalent to that used in Fig. 1G (Materials and Methods; Table S1). The distribution is unchanged (within sampling error).
(PDF)

Table S1 **Numerical screen of large-population model with non-monotonic mutation rates (** **Fig. 1G** **,** **2A–H** **).**
(PDF)

Table S2 **Numerical screen of small-population model with non-monotonic mutation rates (** **Fig. 2I** **).**
(PDF)

Table S3 **Numerical screen of large-population model with “monotonic” mutation rates (** **Figs. 2J, K** **).**
(PDF)

Text S1 **Formulation and analysis of mathematical models (contains supporting figure legends).**
(PDF)
