# Supplementary figures and images for: The Protective Role of Symmetric Stem Cell Division on the Accumulation of Heritable Damage
Source: PLoS Comput Biol. 2014 Aug 14;10(8):e1003802. doi: 10.1371/journal.pcbi.1003802 (PMC4133021; doi:10.1371/journal.pcbi.1003802)

**A**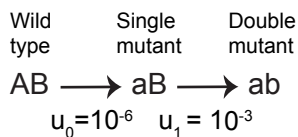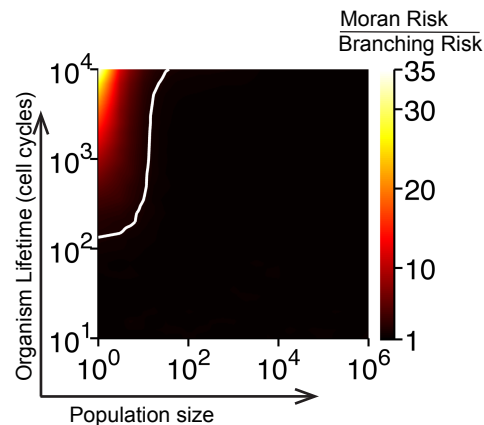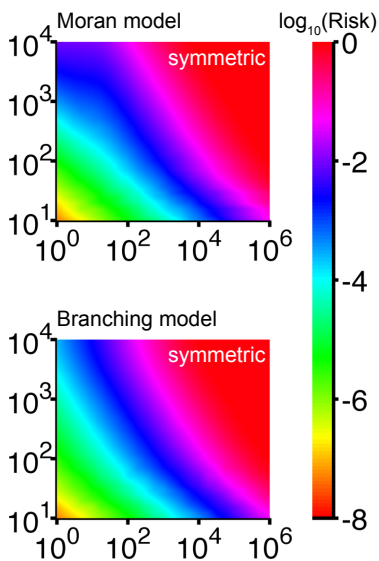**B**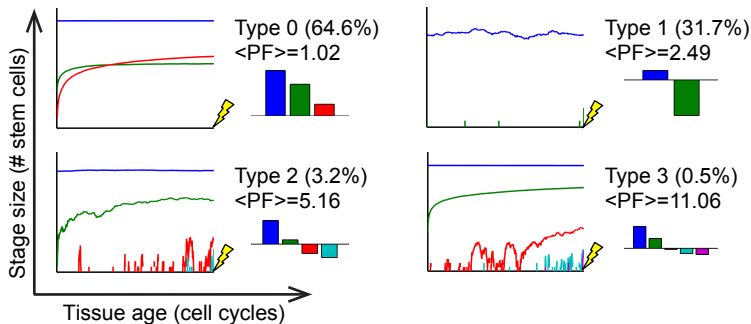

Supplement: Figure S1 — Numerical screen. (A) Concordance of “Moran” and “Branching” models used to screen large and small populations, respectively. The lifetime cumulative risk of accumulating two mutations in a symmetric population was computed for a variety of stem-cell population sizes and organism lifetimes under both models (right panels). The models predict the same cumulative risk over most of parameter space but differ significantly in small populations at large lifetimes, where extinctions of the entire stem cell population in the Branching model reduce risk by at least a factor of two (white contour in left panel). (B) Parameter sets comprising the numerical screen of Table S1 were classified into 4 types based on the number of stochastic stages. Representative symmetric trajectories are shown. Notice the correlation between the number of stochastic stages and mean PF (averaged over all parameter sets with a given number of stochastic stages). (PDF) [file pcbi.1003802.s001.pdf]

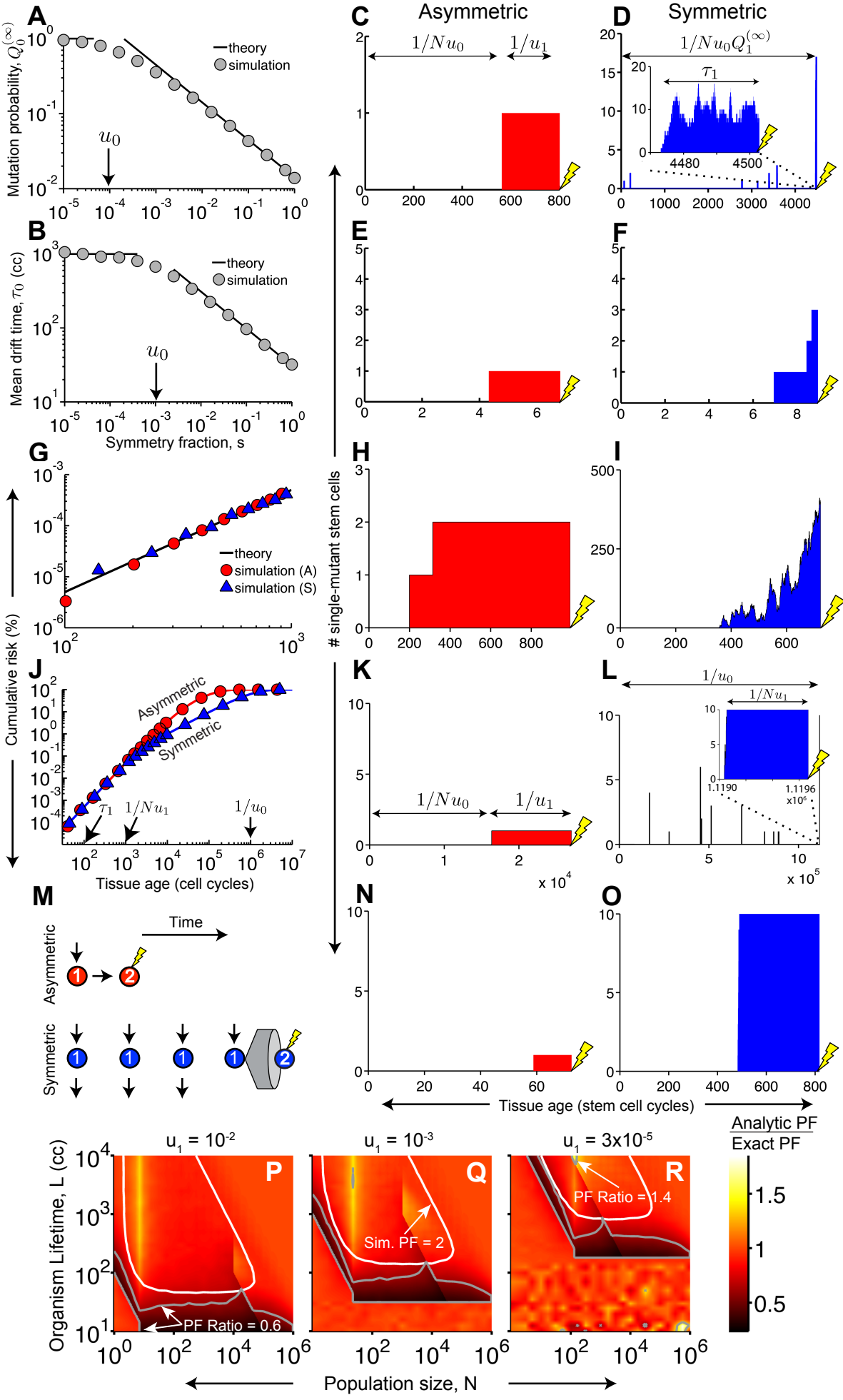

Supplement: Figure S2 — Analysis of stochastic tunneling and sequential fixation regimes. (A, B) A single wild-type stem cell was simulated until either one of its descendants mutated (with probability ) or its lineage extinguished without mutating (with probability ). The mean time that a branching lineage drifts before mutating, , was recorded in those cases where mutation occurred. Panel A shows that the simulated lineage mutation probability (symbols) is well described by Eq. (S24) (lines) whereas panel B shows that the simulated drift time (symbols) is well described by Eq. (S30) (lines). (C–F) Typical dynamics at long times (C, D) and short times (E, F) prior to the production of the first double-mutant stem cell (yellow lightning bolt). Inset to (D) is a magnified view of the last few generations of the simulated dynamics. Population size is N = 103 stem cells and mutation rates are u0 = 10−6 and u1 = 10−3. (G–I) Protection vanishes for small secondary mutation rate, u1≪1/L 2. In these panels, population size is N = 103 stem cells and mutation rates are u0 = 10−6 and u1 = 10−8. (G) Simulated (symbols) and theoretical (line, Eq. (S56)) cumulative risk. (H, I) Typical trajectories that generate a double-mutant stem cell by end of life, T2<L = 103 cc. In both cases, one of the first few single-mutant lineages to arise from the wild-type background produces a double-mutant stem cell that arises improbably early in its parent single-mutant lineage, T2≪≪1/u1. (J–O) Sequential Fixation Regime. In these panels, population size is N = 10 stem cells and mutation rates are u0 = 10−6, u1 = 10−4. (J) Cumulative risk calculated using simulation (symbols) and Eqs. (S48), (S56) and (S59) (lines). (K, N) A double-mutant stem cell typically arises in the first single-mutant stem cell in a purely asymmetric population. (L, O) Dynamics in a purely symmetric population. (L) At long times, t≫1/Nu1, single-mutant lineages frequently extinguish before one survives drift, fixes in the population, and then r [file pcbi.1003802.s002.pdf]

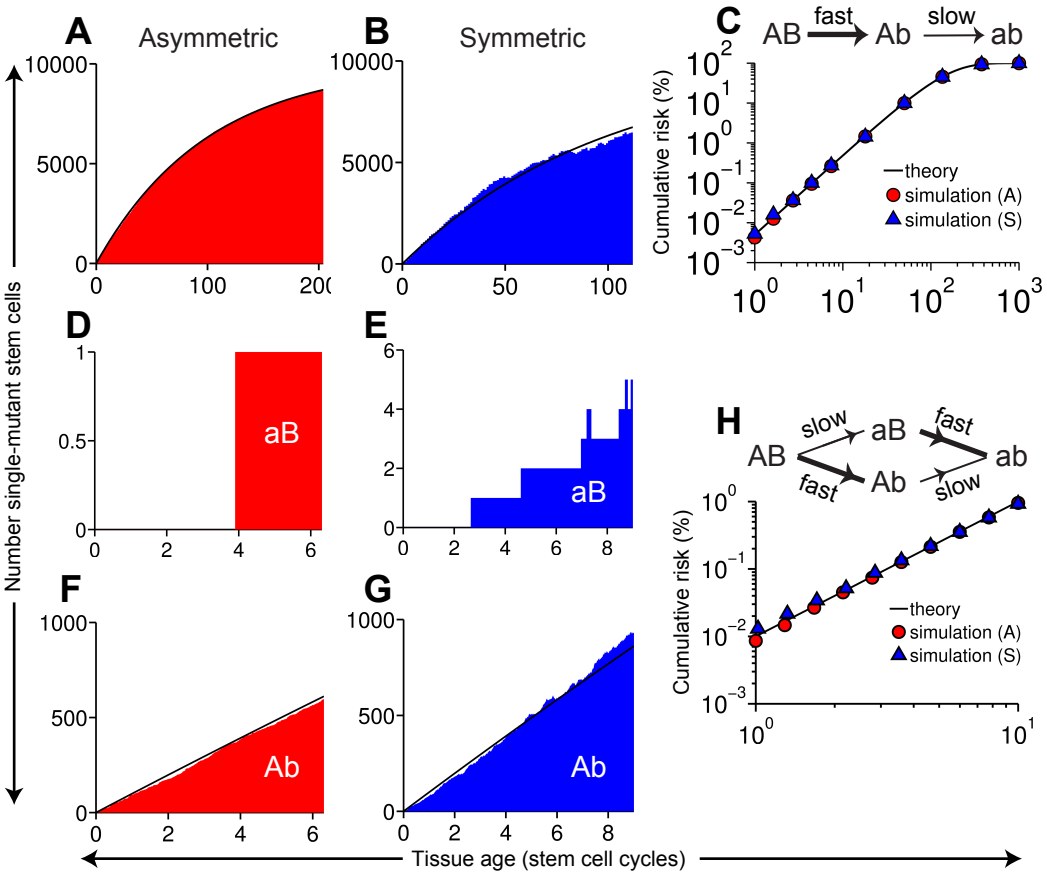

Supplement: Figure S3 — Unordered “fast” and “slow” mutations. (A–C) A “fast-slow” ordered pathway. (A, B) The abundance of Ab stem cells is approximated by its mean value, , which follows from Eq. (S51) when t, 1/u0≪1/u1. (C) Simulated cumulative risk (symbols) is approximated by . (D–H) Dynamics at short times, t≪, of unordered “fast” and “slow” loci. The black line in panels F and G is Eq. (S67). (H) Simulated cumulative risk (symbols) is approximated by Eq. (S71). In all panels, population size is N = 104 stem cells and mutation rates are 10−2 (“fast”) and 10−6 (“slow”). Time courses are plotted until the first double-mutant stem cell appears in the entire stem cell population. (PDF) [file pcbi.1003802.s003.pdf]

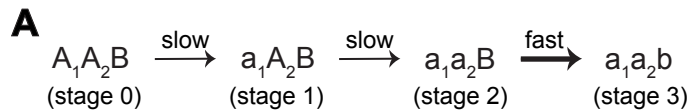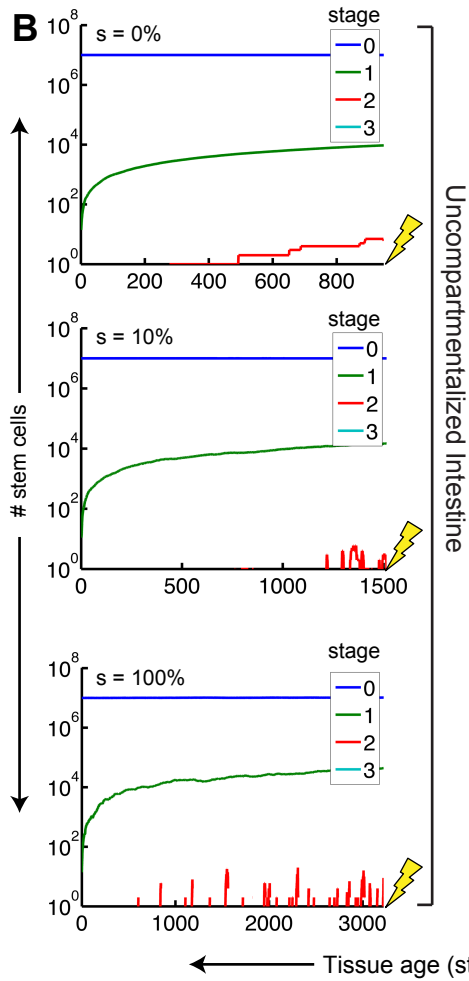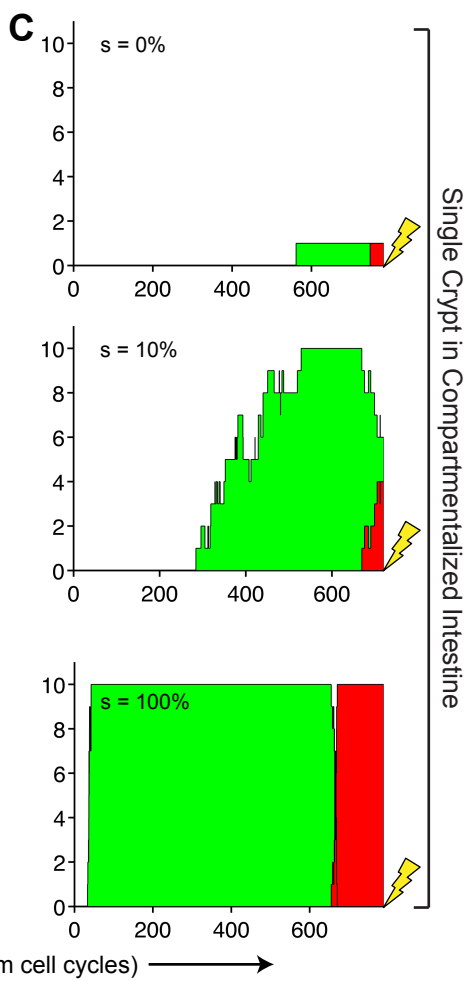

Supplement: Figure S4 — Clonal extinctions out-compete progression in the intestine. (A) Model of mutation accumulation in the intestine. (B) Typical dynamics showing how various patterns of division generate the triple-mutant stem cell in the un-compartmentalized case. (C) Dynamics in a single crypt of a compartmentalized intestine. The purely asymmetric trajectory (s = 0%) is representative of all trajectories examined whereas the mixed (s = 10%) and purely symmetric (s = 100%) trajectories show the most frequently observed type since all four possible combinations of stochastic tunneling and sequential fixation were observed at appreciable frequencies (see also Ref [17] in Text S1). The intestine was assumed to comprise 106 crypts, each containing 10 stem cells. Mutation rates are 10−6 (slow) and 10−3 (fast). (PDF) [file pcbi.1003802.s004.pdf]

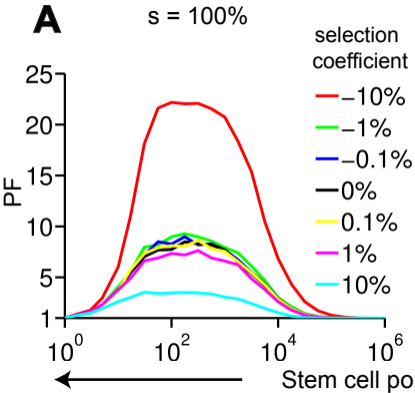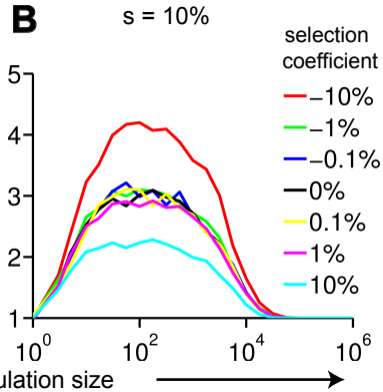

Supplement: Figure S5 — Protection persists when selection acts on stochastic stages. Protection against ordered accumulation of K = 2 mutations after 1000 stem cell cycles for symmetry fractions s = 100% (A) and 10% (B), calculated by Monte Carlo simulation of the generalized model presented in Section 4.1 of Text S1. The selection coefficient is defined in the model by (w1-w0)/w0, where wi is the fitness of stage i (see Section 4.1 of Text S1). The insensitivity of PF to wide variations in the selection coefficient is an example of the general principle in population genetics that selection is ineffective provided the magnitude of the selection coefficient is smaller than the inverse population size. Mutation rates are u0 = 10−6 and u1 = 10−3 per locus per stem cell cycle. (PDF) [file pcbi.1003802.s005.pdf]

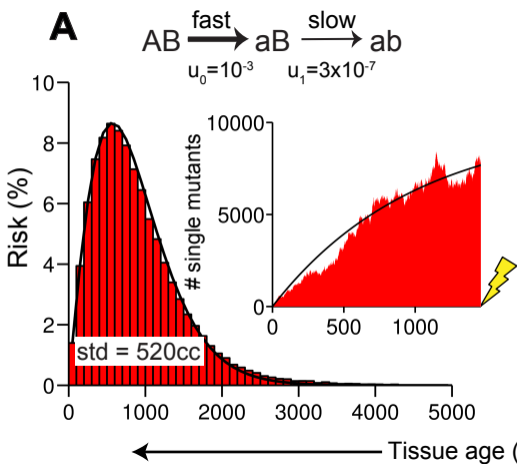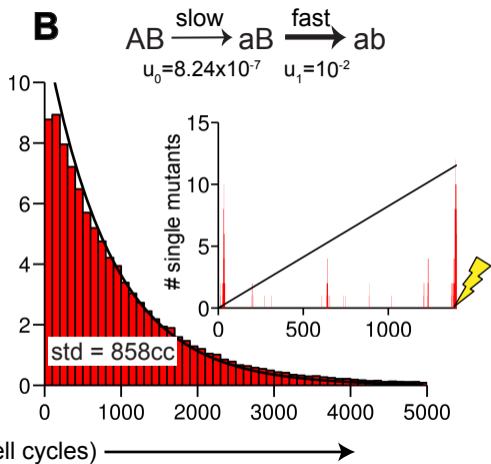

Supplement: Figure S6 — “Increasing” mutation rates yield a broad distribution of latencies. Probability distributions of times at which the first double-mutant stem cell arose in a population of N = 104 symmetrically dividing stem cells, from simulations of the discrete-time branching process defined by Eq. (S1) (bars) and from the probability mass function (lines). In the Deterministic regime (A), the rate constant is given by , where the mean abundance of single-mutant stem cells is , whereas it is in the Stochastic Tunneling regime (B). When the mutation rates are decreasing, the distribution of latency until the first double-mutant stem cell is narrow (A), but when the mutation rates are increasing, the distribution becomes wider (B), even at the same mean latency (858 cell cycles in both cases). Histogram bar height represents the probability that the mutation occurred between the bar edges. Insets show typical stochastic realizations (red) and mean single-mutant abundance (black). (PDF) [file pcbi.1003802.s006.pdf]

1000-fold increase  
in mutation rate

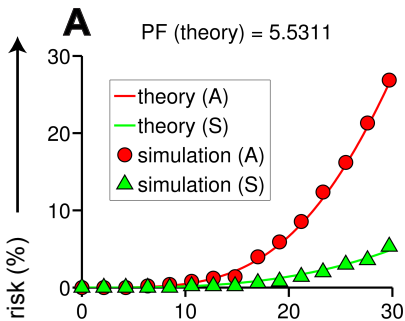

100-fold increase  
in mutation rate

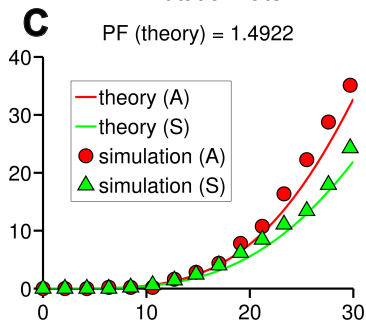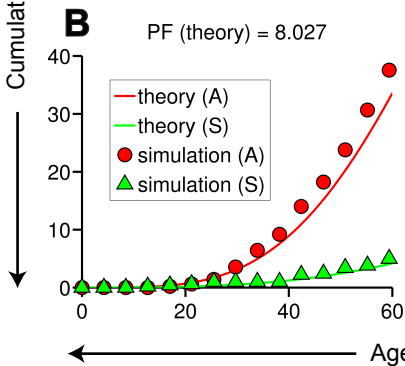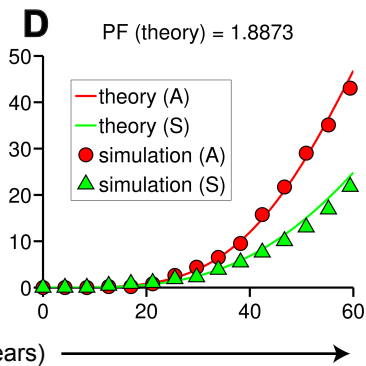

Supplement: Figure S7 — Protection in the human colon. Cumulative risk of ordered accumulation of K = 4 mutations by ages 30 (A, C) and 60 (B, D), assuming mutation rates increase 1000-fold (A, B) or 100-fold (C, D) during the course of mutation accumulation from an initial rate of u0 = 5×10^−7^ per locus per stem cell cycle; see Ref [18] in Text S1. Lines are Eqs. (S75) and (S76) whereas symbols are Monte Carlo simulations (under the Moran model; Section 2.1 of Text S1). The colon is assumed to be compartmentalized into M = 107 crypts, (∼104 crypts/cm2×∼103 cm2/colon; Ref [19] of Text S1) each containing N = 20 stem cells (Ref [20], [21] of Text S1) dividing purely asymmetrically (red) or symmetrically (green) 100 times per year. Mutation rates of consecutive stages (u0; u1; u2; u3) are (A) 5×10^−7^; 5×10^−7^; 5×10^−6^; 5×10^−4^; (B) 5×10^−7^; 5×10^−7^; 5×10^−7^; 5×10^−4^; (C) 5×10^−7^; 5×10^−7^; 5×10^−5^; 5×10^−5^; (D) 5×10^−7^; 5×10^−7^; 5×10^−6^; 5×10^−5^. (PDF) [file pcbi.1003802.s007.pdf]

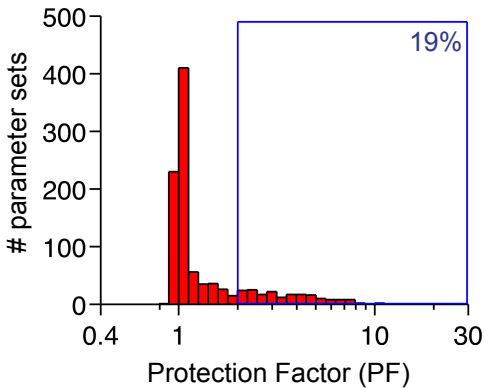

Supplement: Figure S8 — Symmetry protects even when mutations may occur simultaneously in both daughter stem cells. The more accurate ordered mutation accumulation model presented in Section 6 of Text S1 was used to generate a distribution of PFs over a random ensemble of parameter sets equivalent to that used in Fig. 1G (Materials and Methods; Table S1). The distribution is unchanged (within sampling error). (PDF) [file pcbi.1003802.s008.pdf]
