# Supplementary material for: The Protective Role of Symmetric Stem Cell Division on the Accumulation of Heritable Damage
Source: PLoS Comput Biol. 2014 Aug 14;10(8):e1003802. doi: 10.1371/journal.pcbi.1003802 (PMC4133021; doi:10.1371/journal.pcbi.1003802)
Supplement: Table S1 — Numerical screen of large-population model with non-monotonic mutation rates ( Fig. 1G , 2A–H ). (PDF) [file pcbi.1003802.s009.pdf]

Table S1. Numerical screen of large-population model with non-monotonic mutation rates (Fig. 1G, 2A-H)

Parameter	Physical meaning	Range of values
N	Population size	10^4 - 10^9 stem cells
L	Organism lifetime	10^2 - 10^3 cell cycles
u_i; i = 0 … K-1	Mutation rates	10^{-9} - 10^{-2}
K	# accumulated mutations	1 – 10 (chosen uniformly)
