# Supplementary material for: The Protective Role of Symmetric Stem Cell Division on the Accumulation of Heritable Damage
Source: PLoS Comput Biol. 2014 Aug 14;10(8):e1003802. doi: 10.1371/journal.pcbi.1003802 (PMC4133021; doi:10.1371/journal.pcbi.1003802)
Supplement: Table S2 — Numerical screen of small-population model with non-monotonic mutation rates ( Fig. 2I ). (PDF) [file pcbi.1003802.s010.pdf]

Table S2. Numerical screen of small-population model with non-monotonic mutation rates (Fig. 2I)

Parameter	Physical meaning	Range of values
N	Population size	10^0 - 10^3 stem cells
L	Organism lifetime	10^2 - 10^3 cell cycles
u_i; i = 0 … K-1	Mutation rates	10^{-9} - 10^{-2}
K	# accumulated mutations	2
