# Supplementary material for: The Protective Role of Symmetric Stem Cell Division on the Accumulation of Heritable Damage
Source: PLoS Comput Biol. 2014 Aug 14;10(8):e1003802. doi: 10.1371/journal.pcbi.1003802 (PMC4133021; doi:10.1371/journal.pcbi.1003802)
Supplement: Table S3 — Numerical screen of large-population model with “monotonic” mutation rates ( Figs. 2J, K ). (PDF) [file pcbi.1003802.s011.pdf]

Table S3. Numerical screen of large-population model with "monotonic" mutation rates (Figs. 2J, K)

Parameter	Physical meaning	Range of values
N	Population size	10^4 - 10^9 stem cells
L	Organism lifetime	10^2 - 10^3 cell cycles
u_i; i = 0 … k	Early mutation rates	10^{-9} - 10^{-6} (Fig. 2J)<br>10^{-5} - 10^{-2} (Fig. 2K)
u_i; i = k+1 … K-1	Late mutation rates	10^{-5} - 10^{-2} (Fig. 2J)<br>10^{-9} - 10^{-6} (Fig. 2K)
k	Transition stage	0 … K-2 (chosen uniformly)
K	# accumulated mutations	2 – 10 (chosen uniformly)
