# Supplementary material for: The Protective Role of Symmetric Stem Cell Division on the Accumulation of Heritable Damage
Source: PLoS Comput Biol. 2014 Aug 14;10(8):e1003802. doi: 10.1371/journal.pcbi.1003802 (PMC4133021; doi:10.1371/journal.pcbi.1003802)
Supplement: Text S1 — Formulation and analysis of mathematical models (contains supporting figure legends). (PDF) [file pcbi.1003802.s012.pdf]

Text S1:
Formulation and Analysis of Mathematical Models
for
"The protective role of symmetric stem cell division on the accumulation of heritable damage"

Peter T McHale and Arthur D Lander

July 15, 2014

Contents

1 "Branching" model of ordered mutation accumulation 3
1.1 Formulation 3
1.2 Efficient simulation algorithm 4
1.3 Deterministic analysis 5
1.4 Purely asymmetric risk 6
1.5 Heuristic analysis of protection 7
1.5.1 Median time for a stage to extinguish 7
1.5.2 Protection criterion 8
1.6 Probability that a lineage eventually mutates 9
1.7 Mean time that a lineage drifts before mutating 11

2 "Moran" model of ordered mutation accumulation 13
2.1 Formulation 14
2.2 Deterministic analysis 15
2.3 Formal analysis of the accumulation of two mutations 18
2.3.1 Derivation of PF formula 18
2.3.2 Deterministic regime of a purely symmetric population 22

3 Unordered accumulation of two mutations 22
3.1 "Branching" model 22
3.2 "Moran" model 23
3.3 Deterministic solution 24
3.4 Purely asymmetric risk 24
3.5 Purely symmetric risk when loci mutate independently 24

4 Effect of selection on ordered mutation accumulation 25
4.1 Formulation 25
4.2 Deterministic Analysis 26

5 Protection in the compartmentalized intestine 27

6 Effect of allowing for simultaneous mutations in both daughter stem cells 27

7 Supporting References 28

8 Supporting Figure Legends 31

1 "Branching" model of ordered mutation accumulation

We first consider the case in which mutations accumulate in a defined temporal order at a set of loci as shown in Fig. 1E and later (in Section 3) relax this assumption so that mutations may accumulate in any order.

1.1 Formulation

Let S represent a stem cell and D a non-stem cell. Assume the stem cell divisions are synchronized with a fixed cell-cycle duration of unity. When a stem cell divides, it spawns two daughter cells in one of three different ways with probabilities p_r, p_a and p_e:

$$S \longrightarrow \begin{cases} S+S & p_r = \frac{1}{2}s \\ S+D & p_a = 1-s \\ D+D & p_e = \frac{1}{2}s. \end{cases}$$

Here, the fraction of divisions that are symmetric — producing daughter cells with a common fate — is denoted s (not to be confused with a selection coefficient). Numerical homeostasis of the stem-cell pool is ensured on average by balancing the probability of symmetric renewal, p_r, with that of symmetric extinction, p_e. Given a stage-i stem cell, S_i, harboring neutral mutations at $i = 0 \ldots K-1$ loci, each of its daughters independently acquires a mutation at an additional locus, $i+1$, with probability u_i per stem-cell division. Thus, a symmetric renewal gives rise to one of four possible outcomes

$$S_i \longrightarrow \begin{cases} S_i + S_i & (1-u_i)^2 \\ S_i + S_{i+1} & (1-u_i)u_i \\ S_{i+1} + S_i & u_i(1-u_i) \\ S_{i+1} + S_{i+1} & u_i^2. \end{cases}$$

Similarly, an asymmetric division generates mutants according to

$$S_i \longrightarrow \begin{cases} S_i + D_i & (1-u_i)^2 \\ S_i + D_{i+1} & (1-u_i)u_i \\ S_{i+1} + D_i & u_i(1-u_i) \\ S_{i+1} + D_{i+1} & u_i^2, \end{cases}$$

whereas a symmetric differentiation looks like

$$S_i \longrightarrow \begin{cases} D_i + D_i & (1-u_i)^2 \\ D_i + D_{i+1} & (1-u_i)u_i \\ D_{i+1} + D_i & u_i(1-u_i) \\ D_{i+1} + D_{i+1} & u_i^2. \end{cases}$$

Since we restrict our exploration of parameter space to $u_i \leq 10^{-2} \ll 1$, we may neglect probability contributions of order u_i^2 (see Section 6 and Fig. **S8**). Making this approximation,

and recognizing that the outcome $X_i + X_{i+1}$ (where $X = S$ or D) cannot be distinguished from $X_{i+1} + X_i$ in a non-spatial model, we arrive at the following reduced set of outcomes

$$S_i \longrightarrow \begin{cases} S_i + S_i & 1 - 2u_i \\ S_i + S_{i+1} & 2u_i \end{cases}$$

$$S_i \longrightarrow \begin{cases} S_i + D_i & 1 - 2u_i \\ S_i + D_{i+1} & u_i \\ S_{i+1} + D_i & u_i \end{cases}$$

$$S_i \longrightarrow \begin{cases} D_i + D_i & 1 - 2u_i \\ D_i + D_{i+1} & 2u_i. \end{cases}$$

Putting all this together, and book-keeping only the stem cells, we conclude that the division of a stem cell S_i ($i = 0 \ldots K-1$) results in one of five possible outcomes with the following probabilities:

$$S_i \longrightarrow \left\{ \begin{array}{llcl} S_i + S_i & p_{i,r} & = & \frac{1}{2}s(1 - 2u_i) \\ S_i + S_{i+1} & p_{i,rm} & = & \frac{1}{2}s\, 2u_i \\ S_i & p_{i,a} & = & (1-s)(1-u_i) \\ S_{i+1} & p_{i,am} & = & (1-s)u_i \\ \emptyset & p_{i,e} & = & \frac{1}{2}s \end{array} \right\} \tag{S1}$$

This is a discrete-time multi-type branching process [1, 2] in which each stem cell divides independently of all other stem cells. The process starts with N wild-type (stage-0) stem cells and ends when the first stage-K stem cell arises.

1.2 Efficient simulation algorithm

Let $Z_{ij}(t)$ be the (random) number of times that a category-j division in Eq. (S1) occurs at time t in a population of stage-i stem cells of (random) size $N_i(t)$. The stage sizes one cell cycle later are

$$N_0(t+1) = N_0(t) - B_0(t) \tag{S2a}$$
$$N_i(t+1) = N_i(t) + A_{i-1}(t) - B_i(t) \qquad i = 1, \ldots, K-1 \tag{S2b}$$
$$N_K(t+1) = N_K(t) + A_{K-1}(t) \tag{S2c}$$

where

$$\begin{aligned} A_i(t) &= Z_{i,rm}(t) + Z_{i,am}(t) \\ B_i(t) &= Z_{i,am}(t) + Z_{i,e}(t) - Z_{i,r}(t). \end{aligned}$$

Notice that we have assumed that the stage-K stem cell has no birth-death dynamics. Instead $S_K \to S_K$ at each cell cycle. This simplification does not affect the conclusions we draw since the statistics that we compute relate to the appearance of the first stage-K stem cell and are thus independent of its dynamics.

Conditioned on the sizes of the various stages, $\{N_i(t) = n_i\}$, the number of divisions in each category, $\vec{Z}_i = (Z_{i,r}\,, Z_{i,rm}\,, Z_{i,a}\,, Z_{i,am}\,, Z_{i,e})$, is distributed according to the multinomial

distribution [3],

$$P[\vec{Z}_i(t) = \vec{z} \mid N_i(t) = n_i] = \frac{n_i!}{\prod_j z_j!} \prod_j p_{ij}^{z_j} \;, \tag{S3}$$

where $\sum_j z_j = n_i$. This is an efficient way to simulate the stochastic process.

1.3 Deterministic analysis

Averaging the random variables in Eq. (S2) gives rise to terms of the form $\langle Z_{ij}(t)\rangle$ that can be simplified by conditioning on the stage size as follows

$$\begin{aligned}\langle Z_{ij}(t)\rangle &= \sum_{\vec{z}} z_j \, P[\vec{Z}_i(t) = \vec{z}] \\ &= \sum_{n_i} \left\{ \sum_{\vec{z}} z_j \, P[\vec{Z}_i(t) = \vec{z} \mid N_i(t) = n_i] \right\} P[N_i(t) = n_i] \,.\end{aligned}$$

The factor in curly brackets is the average number of times that a category-j division occurs in a fixed-size population of stage-i stem cells and is given by the product of the stage size, n_i, and the probability of a stem cell executing a category-j division, p_{ij}. Making this replacement in the curly brackets immediately yields

$$\langle Z_{ij}(t)\rangle = \langle N_i(t)\rangle \, p_{ij}.$$

Using this result and the expressions for the category probabilities p_{ij} given in Eq. (S1), one may then show that the average population sizes obey

$$\langle N_0(t+1)\rangle - \langle N_0(t)\rangle = -\langle N_0(t)\rangle \, u_0 \tag{S4a}$$
$$\langle N_i(t+1)\rangle - \langle N_i(t)\rangle = \langle N_{i-1}(t)\rangle \, u_{i-1} - \langle N_i(t)\rangle \, u_i \qquad i = 1, \ldots, K-1 \tag{S4b}$$
$$\langle N_K(t+1)\rangle - \langle N_K(t)\rangle = \langle N_{K-1}(t)\rangle \, u_{K-1} \tag{S4c}$$

After a few cell cycles, $t \gg 1$, these equations are well approximated by their continuous-time form

$$d\langle N_0\rangle/dt = -\langle N_0\rangle \, u_0 \tag{S5a}$$
$$d\langle N_i\rangle/dt = \langle N_{i-1}\rangle \, u_{i-1} - \langle N_i\rangle \, u_i \qquad i = 1, \ldots, K-1 \tag{S5b}$$
$$d\langle N_K\rangle/dt = \langle N_{K-1}\rangle \, u_{K-1} \tag{S5c}$$

If the mutation rates are identical and equal to u, then the relative abundance of the stages, $\langle N_i\rangle/N$, follows a truncated Poisson distribution

$$\begin{aligned}\langle N_i\rangle/N &= (ut)^i e^{-ut}/i! \qquad i = 0, \ldots, K-1 \\ \langle N_K\rangle/N &= 1 - \textstyle\sum_{i=0}^{K-1} \langle N_i\rangle/N.\end{aligned}$$

We can also make analytical progress when the mutation rates are not all equal. Initially, the dynamics of each stage is determined solely by the influx from the previous stage,

$$d\langle N_0\rangle/dt = 0 \tag{S6a}$$
$$d\langle N_i\rangle/dt = \langle N_{i-1}\rangle u_{i-1} \qquad i = 1,\dots,K \tag{S6b}$$

yielding

$$\langle N_0\rangle = N \tag{S7a}$$
$$\langle N_i\rangle = N u_0 \dots u_{i-1} t^i / i! \qquad i = 1\dots K. \tag{S7b}$$

Using these solutions we may derive the following expression for the influx to stage i

$$\langle N_{i-1}\rangle u_{i-1} = \langle N_i\rangle i/t \qquad i = 1\dots K. \tag{S8}$$

Plugging this expression into the influx-outflux inequality $\langle N_{i-1}\rangle u_{i-1} \gg \langle N_i\rangle u_i$ shows that the solutions in Eq. (S7) are valid provided

$$t \ll 1/u_0 \tag{S9a}$$
$$t \ll i/u_i \qquad i = 1\dots K \tag{S9b}$$

which is met for most biologically realistic parameter values. Combining Eqs. (S8) and (S9) implies that the stage abundances are monotonically decreasing

$$\langle N_i\rangle \ll \langle N_{i-1}\rangle \qquad i = 1\dots K. \tag{S10}$$

1.4 Purely asymmetric risk

In a tissue undergoing a purely asymmetric pattern of division ($s = 0$), each stem cell behaves independently of all the others. This has two implications. First, the probability that the first K-fold mutant stem cell arises by time t, $R_K(t) = P[\,T_K < t\,]$, is related to the corresponding risk per stem cell, $r_K(t)$, by

$$R_K = 1 - (1 - r_K)^N\,,$$

where N is the population size. Second, the average number of stage-K stem cells is

$$\langle N_K\rangle = r_K N.$$

Putting these formulae together, we get

$$R_K = 1 - \left(1 - \frac{\langle N_K\rangle}{N}\right)^N. \tag{S11}$$

Keeping $\langle N_K\rangle$ constant while taking the limit $N \to \infty$ yields

$$R_K = 1 - e^{-\langle N_K\rangle}. \tag{S12}$$

This formula, together with Eqs. (S5), was used to screen for parameter sets with purely asymmetric lifetime risks in a defined range.

1.5 Heuristic analysis of protection

In this section we first derive the time scale on which a population of stem cells undergoing neutral drift is driven to extinction. We then use this result to heuristically derive the conditions under which symmetry is expected to delay mutation accumulation.

1.5.1 Median time for a stage to extinguish

Suppose that we observe n stem cells in a particular stage i of a purely symmetric population at time $t = 0$. Prior to mutation the stem-cell dynamics may be approximated by the continuous-time branching process,

$$S_i \longrightarrow \begin{cases} S_i + S_i & \text{prob} = \frac{1}{2} \\ \emptyset & \text{prob} = \frac{1}{2} \end{cases} ,$$

with average cell-cycle time of unity. The distribution of times, T_e, at which the stage extinguishes is therefore [4, Eq. 8.58]

$$\begin{aligned} P[\,T_e < t \,|\, N_i(0) = n\,] &= P[\,N_i(t) = 0 \,|\, N(0) = n\,] \\ &= \left(\frac{\frac{1}{2}t}{1+\frac{1}{2}t}\right)^n, \end{aligned} \tag{S13}$$

where $N_i(t)$ is the random number of stage-i stem cells at time t. Scaling time according to $\hat{t} = t/2n$, the distribution takes on the form

$$P[\,\hat{T}_e < \hat{t}\,] = \frac{1}{\left(1 + \frac{1/\hat{t}}{n}\right)^n}.$$

Keeping $\hat{t}$ constant while taking the limit $n \to \infty$ yields the Fréchet distribution from Extreme Value Theory [5, p. 9],

$$P[\,\hat{T}_e < \hat{t}\,] = e^{-1/\hat{t}}.$$

An extreme-value distribution is expected since T_e is the maximum of the set of independent and identically distributed extinction times corresponding to the n lineages (clones) comprising the initial population. The Fréchet distribution has a rather heavy right tail, $1 - e^{-2n/t} \sim 2n/t$ as $t \to \infty$, implying that the mean extinction time diverges logarithmically. We therefore used the median time to extinction, defined by

$$P[\,T_e < \tau_e\,] = \frac{1}{2},$$

and given by

$$\tau_e = \frac{2n}{\ln 2}, \tag{S14}$$

as a measure of the time needed for a stage to extinguish given that it is observed to contain n stem cells. This expression is asymptotically exact at large stage sizes whereas it is a sufficiently good approximation for our purposes at small sizes. (The exact value implied by Eq. (S13) is $2^{1-1/n}/\left(1-2^{-1/n}\right)$.)

1.5.2 Protection criterion

We first determine which lineage (clone) of stage-i stem cells is responsible for progression to the next stage during the organism's lifetime, L. Eq. (S6b), which is valid provided $L \ll i/u_i$ (see Eq. (S9)), may be re-cast as

$$\langle N_i(L)\rangle \approx \textstyle\int_0^L \langle N_{i-1}(t)\rangle\, u_{i-1}\, dt \qquad i = 1 \ldots K-1$$

which says that the average lifetime abundance of stage-i stem cells approximately equals the average number of stage-i lineages founded during the course of life. In a symmetric population, most of the lineages never reach a substantial size and extinguish within a few cell cycles (Fig. 2D). Since each of these lineages has probability of order $1/x$ of reaching size $x \geq 1$ or larger [6], on average only one of them will reach a size of at least $\langle N_i(L)\rangle \geq 1$. Thus the mean lifetime abundance approximately measures the size of the largest clone likely to occur during a lifetime (asterisk in Fig. 2D). By virtue of its size, this clone carries the majority of the risk of progression to the next stage [6]. This intuitive picture fails when $\langle N_{i+1}(L)\rangle \ll 1$ because progression then occurs in rare lineages that grow to sizes much larger than $\langle N_i(L)\rangle$ (see Section 2.3.1 and Fig. **S2**I).

Having identified the clone most susceptible to progression, we next determine the circumstances under which symmetric divisions delay its progression to the next stage. If the clone were dividing purely asymmetrically, it would progress in a time of order $1/\langle N_i(L)\rangle\, u_i$. However, since it is dividing purely symmetrically, it will extinguish in a time of order $\langle N_i(L)\rangle$ (derived in Section 1.5.1). Symmetric extinctions ought to delay progression provided the clone extinguishes faster than it progresses, $\langle N_i(L)\rangle \ll 1/\langle N_i(L)\rangle\, u_i$. Thus the criterion for symmetry-dependent protection of stage i is

$$\langle N_i(L)\rangle \ll 1/\sqrt{u_i} \qquad i = 1 \ldots K-1 \tag{S15}$$

and is valid provided $L \ll i/u_i$ and $\langle N_{i+1}(L)\rangle \geq 1$ (notice that the last condition implies $\langle N_i(L)\rangle \geq 1$ by virtue of Eq. (S10)). The threshold population size, $1/\sqrt{u_i}$, represents the size a symmetric stage-i lineage needs to reach in order to progress to the next stage [6]. Re-casting Eq. (S8) in the form

$$\langle N_i(L)\rangle \approx (i+1)\,\langle N_{i+1}(L)\rangle\,/u_i L \qquad i = 0 \ldots K-1 \tag{S16}$$

and plugging this expression for the abundance into Eq. (S15), we arrive at an alternate

form of the protection condition

$$u_i \gg \left(\, (i+1) \, \langle N_{i+1}(L) \rangle \, / L \, \right)^2 \qquad\qquad i = 1 \ldots K-1 \tag{S17}$$

again valid for $L \ll i/u_i$ and $\langle N_{i+1}(L) \rangle \geq 1$. Eqs. (S15) and (S17) suggest natural scales on which to measure abundance and mutation rate, respectively,

$$\overline{n}_i = \langle N_i(L) \rangle \sqrt{u_i} \tag{S18a}$$

$$\overline{u}_i = u_i \left(\, L/(i+1) \, \langle N_{i+1}(L) \rangle \, \right)^2 . \tag{S18b}$$

Eq. (S16) may then be used to establish the following simple relation between the scaled parameters

$$\overline{u}_i = \frac{1}{\overline{n}_i^2} \tag{S19}$$

valid for $L \ll i/u_i$.

1.6 Probability that a lineage eventually mutates

Given that there is initially just one wild-type (stage-0) stem cell, we wish to calculate the probability

$$\begin{aligned} Q_0^{(\infty)} &= \lim_{t \to \infty} Q_0(t) \\ &= \lim_{t \to \infty} (1 - P_0(t)) \end{aligned}$$

that eventually one of its descendants mutates, where $P_0(t)$ is the probability of surviving the mutation by time t. Since the stage-1 stem cell has no dynamics ($K = 1$), the event $(N_1(t) = 0)$ is equivalent to the nonoccurrence of the mutation by time t,

$$P_0(t) = P[\, N_1(t) = 0 \,|\, N_0(0) = 1, N_1(0) = 0 \,], \tag{S20}$$

where $N_0(t)$ and $N_1(t)$ denote the numbers of wild-type and mutant stem cells, respectively.

To carry out the calculation we first need to introduce the theory of multi-type branching processes. Using the shorthand $(n_0, n_1; t)$ to represent the event $(N_0(t) = n_0, N_1(t) = n_1)$, we define

$$\begin{aligned} F_0(x_0, x_1; t) &= \sum_{n_0, n_1} P[\, n_0, n_1; t \,|\, 1, 0; 0 \,] \, x_0^{n_0} \, x_1^{n_1} \\ F_1(x_0, x_1; t) &= \sum_{n_0, n_1} P[\, n_0, n_1; t \,|\, 0, 1; 0 \,] \, x_0^{n_0} \, x_1^{n_1} . \end{aligned}$$

The probability generating functions, F_0 and F_1, satisfy the recursion relations [1, p. 114][7, p. 406][8, p. 116]

$$F_0(t+1) = f_0 \left(F_0(t), F_1(t) \right) \tag{S21a}$$

$$F_1(t+1) = f_1 \left(F_0(t), F_1(t) \right), \tag{S21b}$$

where we have dropped the arguments (x_0, x_1) for clarity, and

$$\begin{aligned} f_0(x_0,x_1) &= F_0(x_0,x_1;1) &= p_{0,r}\, x_0^2 + p_{0,rm}\, x_0\, x_1 + p_{0,a}\, x_0 + p_{0,am}\, x_1 + p_{0,e} \\ f_1(x_0,x_1) &= F_1(x_0,x_1;1) &= x_1 \end{aligned}$$

are the probability generating functions evaluated at the first division.

Having introduced the theory of multi-type branching processes, we now see that the probability that the lineage descending from a stage-0 stem cell survives a mutation is just $P_0(t) = F_0(1, 0; t)$. Using the recursion relations, Eq. (S21), we deduce that

$$P_0(t+1) \;=\; f_0\left(P_0(t), 0\right),$$

where the second argument of f_0 is

$$F_1(1,0;t) = P\left[\, N_1(t) = 0 \,|\, N_0(0) = 0, N_1(0) = 1\,\right] = 0$$

since the mutant cannot extinguish. We may rewrite the kinetic equation for $P_0(t)$ as

$$P_0(t+1) - P_0(t) = p_{0,r}\, P_0(t)^2 - (1 - p_{0,a})\, P_0(t) + p_{0,e}. \tag{S22}$$

The long-time limit, $P_0^{(\infty)} = \lim_{t\to\infty} P_0(t)$, therefore satisfies the quadratic equation

$$p_{0,r}\, x^2 - (1 - p_{0,a})\, x + p_{0,e} = 0. \tag{S23}$$

The physically meaningful solution is given by the smallest root and can be written in the form

$$P_0^{(\infty)} = \alpha_0 - \sqrt{\alpha_0^2 - \beta_0},$$

where

$$\begin{aligned} \alpha_0 &= 1 + \beta_0\gamma_0 \\ \beta_0 &= \frac{1}{1 - 2u_0} \\ \gamma_0 &= \frac{u_0}{s} + u_0. \end{aligned}$$

It is convenient in the analysis that follows to re-write the expression under the square root as

$$\alpha_0^2 - \beta_0 = \frac{2u_0}{s}\beta_0 + \left(\beta_0\gamma_0\right)^2.$$

Biologically plausible values for the mutation rate satisfy $u_0 \ll 1$, in which case $\beta_0 \approx 1$ and

$$P_0^{(\infty)} \approx 1 + \gamma_0 - \sqrt{\frac{2u_0}{s} + \gamma_0^2},$$

If the symmetry fraction is small compared with the mutation rate, $s \ll u_0$, then $\gamma_0 \approx \frac{u_0}{s}$,

and the square root is approximated by $1 + \frac{u_0}{s}$, so that the lineage mutation probability is

$$Q_0^{(\infty)} \approx 1.$$

In this regime, the lineage behaves as if it were dividing purely asymmetrically. Now consider the alternative limit where the symmetry fraction is large compared with the mutation rate, $s \gg u_0$. There are two sub-cases: $s \sim 1$ and $s \ll 1$. In both cases γ_0 is of the same order as $2u_0/s$ so that the leading-order term in the lineage mutation probability is

$$Q_0^{(\infty)} \approx \sqrt{\frac{2u_0}{s}}.$$

Generally, the probability that the lineage descending from a stage-i stem cell accumulates one further mutation is (Fig. **S2**(A))

$$Q_i^{(\infty)} = \begin{cases} 1 & s \ll u_i \\ \sqrt{\frac{2u_i}{s}} & s \gg u_i. \end{cases} \tag{S24}$$

Conceptually similar manipulations can be employed to calculate the probability that a lineage accumulates two or more mutations (see also [6]).

1.7 Mean time that a lineage drifts before mutating

Given that the lineage descending from a wild-type (stage-0) stem cell picks up a mutation, what is the mean time it drifts before doing so, τ_0? Remembering that stage-1 stem cells have no dynamics, we may write

$$\tau_0 = \langle\, T_1 \,|\, N_0(0) = 1, N_1(0) = 0, N_1(\infty) > 0 \,\rangle\,,$$

where T_1 is a random variable representing the time at which the first single-mutant stem cell arises in the lineage. The mean drift time τ_0 is related to the conditional probability of surviving the mutation, $P[\,T_1 > t \,|\, N_1(\infty) > 0\,] = P[\,N_1(t) = 0 \,|\, N_1(\infty) > 0\,]$, by

$$\tau_0 = \sum_{t=0}^{\infty} P[\,N_1(t) = 0 \,|\, N_1(\infty) > 0\,], \tag{S25}$$

where the conditioning on $N_0(0) = 1, N_1(0) = 0$ has been made implicit in the interests of clarity. The summand is related to the unconditioned survival probability in Eq. (S20) by

$$P_0(t) = P_0^{(\infty)} + P[\,N_1(t) = 0 \,|\, N_1(\infty) > 0\,] \left(1 - P_0^{(\infty)}\right).$$

Using this relation in Eq. (S25), and replacing the sum by an integral (a valid approximation for large drift times, $\tau_0 \gg 1$), the mean time until the lineage mutates becomes

$$\tau_0 = \int_0^\infty \frac{P_0(t) - P_0^{(\infty)}}{1 - P_0^{(\infty)}}\, dt\,. \tag{S26}$$

The survival probability $P_0(t)$ satisfies a Riccati equation,

$$dP_0/dt = p_{0,r}\, P_0^2 - (1 - p_{0,a})\, P_0 + p_{0,e}\,,$$

which is the continuous-time approximation to Eq. (S22). The time-dependent solution is

$$P_0 = \frac{P_0^{(\infty)} - r\theta\phi}{1 - \theta\phi}, \tag{S27}$$

where

$$\begin{aligned} \theta &= e^{-\left(r - P_0^{(\infty)}\right)p_{0,r}t} \\ \phi &= \frac{1 - P_0^{(\infty)}}{1 - r} \end{aligned}$$

and $P_0^{(\infty)}$ and r are the two roots of Eq. (S23). As shown in Section 1.6, the roots may be approximated by

$$\begin{aligned} P_0^{(\infty)} &\approx 1 + \gamma_0 - \sqrt{\frac{2u_0}{s} + \gamma_0^2} \\ r &\approx 1 + \gamma_0 + \sqrt{\frac{2u_0}{s} + \gamma_0^2}\,, \end{aligned}$$

where, as in Section 1.6,

$$\gamma_0 = \frac{u_0}{s} + u_0.$$

If the symmetry fraction is small, $s \ll u_0$, then $P_0^{(\infty)} \approx 0$ (see Section 1.6), $r \approx 2u_0/s \gg 1$, and the survival probability is approximated by its purely asymmetric form,

$$P_0(t) = e^{-u_0 t}. \tag{S28}$$

Performing the integral in Eq. (S26) yields

$$\tau_0 = \frac{1}{u_0},$$

which is indeed large, $\tau_0 \gg 1$. If, on the other hand, the symmetry fraction is large, $s \gg u_0$, then

$$P_0^{(\infty)} \approx 1 - \sqrt{\frac{2u_0}{s}}$$
$$r \approx 1 + \sqrt{\frac{2u_0}{s}},$$

(see Section 1.6). After some algebra, Eq. (S26) reduces to

$$\tau_0 = \int_0^\infty \frac{2\theta}{1+\theta}\, dt\,,$$

where

$$\theta \approx e^{-\sqrt{2u_0 s}\, t}.$$

Performing the integral yields

$$\tau_0 = \frac{2\ln 2}{\sqrt{2u_0 s}},$$

which is again large, $\tau_0 \gg 1$. We note that at small times, $t \ll \tau_0$, we have $\theta \approx 1 - \sqrt{2u_0 s}\, t$, so that the survival probability, Eq. (S27), reduces to $P_0(t) \approx 1 - u_0 t$, which, Eq. (S28) tells us, is also the short-time form in the limit of small symmetry fractions, $s \ll u_0$. In other words, the probability that a lineage mutates by time $t \ll \tau_0$ is given by

$$Q_0(t) \approx u_0 t, \tag{S29}$$

irrespective of the value of the symmetry fraction.

Generally, given that the lineage descending from a stage-i stem cell picks up an additional mutation, the mean time it takes to do so is (Fig. **S2**(B))

$$\tau_i = \begin{cases} \frac{1}{u_i} & s \ll u_i \\ \frac{2\ln 2}{\sqrt{2u_i s}} & s \gg u_i. \end{cases} \tag{S30}$$

2 "Moran" model of ordered mutation accumulation

The total population size fluctuates under the branching model. These fluctuations are relatively insignificant in large populations but can significantly violate numerical homeostasis in small populations. Yet real tissues attenuate errant fluctuations via feedback mechanisms. We therefore developed a phenomenological model of feedback that captures all of the stem cell divisions defined by Eq. (S1). This model is a variation on the well-known Moran model from population genetics [9; 10, p. 79] in which time is continuous and the cell cycle period is exponentially distributed with a mean value of unity.

2.1 Formulation

We define first how the asymmetric divisions are treated in the model. The rate at which asymmetric divisions occur in a population of stem cells is $N(1-s)$, where N is the time-invariant total number of stem cells and s is the probability that a stem cell division is symmetric. When such a division occurs, it occurs in a stage-i stem cell with probability n_i/N, where n_i is the number of stage-i stem cells. Only asymmetric divisions in which the daughter mutates need to be simulated since only those change the state of the population — decrementing the number of stage-i stem cells while incrementing the number of stage-$(i+1)$ stem cells. The net rates of such events are

$$\lambda_i^{(am)} = (1-s)n_i u_i \qquad i = 0 \ldots K-1. \tag{S31}$$

We turn next to the symmetric divisions. In the Moran approach, each population-incrementing symmetric renewal is accompanied by a population-decrementing symmetric extinction. Together these divisions constitute one replacement event. Balancing renewals with extinctions in this way ensures that the net population size is strictly constant. Since each replacement constitutes two stem cell divisions, replacements in a purely symmetric population must occur at half the rate of stem cell divisions in an equivalent purely asymmetric population. Thus the rate of replacements in a population undergoing a mix of replacements and asymmetric divisions is $N\frac{1}{2}s$. When a replacement occurs, a stage-i stem cell is chosen for renewal with probability n_i/N whereas a stage-j stem cell is chosen for extinction with probability n_j/N. The symmetric renewal results in mutation of one of the two stem cell daughters with probability $2u_i$, in which case the number of stage-$(i+1)$ stem cells is incremented whereas the number of stage-j stem cells is decremented. The case $i+1=j$ need not be simulated since it does not change the state of the population. The net rates of such events are

$$\lambda_{ij}^{(sm)} = \tfrac{1}{2}sn_i 2u_i \tfrac{n_j}{N}\left(1-\delta_{i+1,j}\right) \qquad i,j = 0 \ldots K-1. \tag{S32}$$

Alternatively, with probability $1-2u_i$, the symmetric renewal is of the form $S_i \to S_i + S_i$, in which case stage-i is incremented whereas stage-j is decremented. Again the case $i=j$ need not be simulated. The net rates of these events are

$$\lambda_{ij}^{(s)} = \tfrac{1}{2}sn_i(1-2u_i)\tfrac{n_j}{N}\left(1-\delta_{i,j}\right) \qquad i,j = 0 \ldots K-1. \tag{S33}$$

This process is simulated by choosing the time interval until the next event by sampling from an exponential distribution with intensity equal to

$$\sum_{i=0}^{K-1} \lambda_i^{(am)} + \sum_{i,j=0}^{K-1} \left(\lambda_{ij}^{(sm)} + \lambda_{ij}^{(s)}\right)$$

and executing one of the events described above with probability proportional to its rate (the Gillespie algorithm). As expected, it coincides with the branching model at long times and large population sizes (Fig. **S1**A).

2.2 Deterministic analysis

We first derive the deterministic equations for a general continuous-time stochastic process. Letting $(\vec{n}, t)$ denote the event $(\vec{N}(t) = \vec{n})$, the expected size of the stage-i stem cell population is

$$\begin{aligned} \langle N_i(t+\delta t)\rangle &= \sum_{\vec{n}'} n_i' P(\vec{n}', t+\delta t) \\ &= \sum_{\vec{n}} \left\{ \sum_{\vec{n}'} n_i'\, p_{\vec{n}\to\vec{n}'} \right\} P(\vec{n}, t), \end{aligned} \tag{S34}$$

with transition probability

$$p_{\vec{n}\to\vec{n}'} = P(\vec{n}', t+\delta t \,|\, \vec{n}, t).$$

It is useful to decompose the term in curly brackets as follows

$$\sum_{\vec{n}'} n_i'\, p_{\vec{n}\to\vec{n}'} = n_i \left[\sum_{\vec{n}':n_i'=n_i} p_{\vec{n}\to\vec{n}'} \right] + \sum_{\vec{n}':n_i'\neq n_i} n_i'\, p_{\vec{n}\to\vec{n}'}. \tag{S35}$$

Since the system must transition to some state, we may cast the term in square brackets as

$$\sum_{\vec{n}':n_i'=n_i} p_{\vec{n}\to\vec{n}'} = 1 - \sum_{\vec{n}':n_i'\neq n_i} p_{\vec{n}\to\vec{n}'}.$$

Inserting this expression into Eq. (S35) and rearranging we get

$$\sum_{\vec{n}'} n_i'\, p_{\vec{n}\to\vec{n}'} = n_i + \sum_{\vec{n}':n_i'\neq n_i} (n_i' - n_i)\, p_{\vec{n}\to\vec{n}'}.$$

Plugging this into Eq. (S34), dividing across by δt, and taking the limit $\delta t \to 0$, we arrive at an equation for the mean size of the stage-i stem cell population

$$d\langle N_i(t)\rangle/dt = \sum_{\vec{n}} \Lambda_{\vec{n}}^{(i)}\, P(\vec{n}, t), \tag{S36}$$

where

$$\begin{aligned} \Lambda_{\vec{n}}^{(i)} &= \sum_{\vec{n}':n_i'\neq n_i} (n_i' - n_i)\, \lambda_{\vec{n}\to\vec{n}'} \\ \lambda_{\vec{n}\to\vec{n}'} &= \lim_{\delta t\to 0} p_{\vec{n}\to\vec{n}'}/\delta t. \end{aligned} \tag{S37}$$

Having derived a general equation governing the time dependence of the mean population sizes, we next apply it to the stochastic process described in Section 2.1. Given a vector $\vec{n} = (n_0, n_1 \ldots n_K)$ describing the numbers of stage-$0, 1 \ldots K$ stem cells in a population, Table 1 classifies the states $\vec{n}' = (n_0', n_1' \ldots n_K')$ that can be reached in a single transition and that have $n_i' \neq n_i$. Inserting these state transitions into Eq. (S37) reduces it to

$\lambda_{\vec{n}\to\vec{n}'}$	$n_i' - n_i$	i
$\lambda_{i-1}^{(am)}$	$+1$	$1 \ldots K$
$\lambda_{i}^{(am)}$	-1	$0 \ldots K-1$
$\lambda_{i-1,j}^{(sm)}$	$+1$	$1 \ldots K$
$\lambda_{j,i}^{(sm)}$	-1	$0 \ldots K-1$
$\lambda_{i,j}^{(s)}$	$+1$	$0 \ldots K-1$
$\lambda_{j,i}^{(s)}$	-1	$0 \ldots K-1$

Table 1: Classification of state transitions that change the number of stage-i stem cells. The transition rates appearing in the first column are defined in Section 2.1. The allowed range of j is $0 \ldots K-1$ in all cases.

$$\Lambda_{\vec{n}}^{(i)} = \left\{ \begin{array}{ll} -\lambda_0^{(am)} + \sigma_0^{(s)} - \Sigma_0 & i = 0 \\ \lambda_{i-1}^{(am)} - \lambda_i^{(am)} + \sigma_{i-1}^{(sm)} + \sigma_i^{(s)} - \Sigma_i & i = 1 \ldots K-1 \\ \lambda_{K-1}^{(am)} + \sigma_{K-1}^{(sm)} & i = K \end{array} \right\} \tag{S38}$$

where

$$\sigma_{i-1}^{(sm)} = \sum_{j=0}^{K-1} \lambda_{i-1,j}^{(sm)} \tag{S39a}$$

$$\sigma_{i}^{(s)} = \sum_{j=0}^{K-1} \lambda_{i,j}^{(s)} \tag{S39b}$$

$$\Sigma_i = \sum_{j=0}^{K-1} \left(\lambda_{j,i}^{(sm)} + \lambda_{j,i}^{(s)} \right) \tag{S39c}$$

The summation in Eq. (S39a) can be performed immediately

$$\begin{aligned} \sigma_{i-1}^{(sm)} &= \frac{1}{2} s n_{i-1}\, 2u_{i-1} \sum_{j=0}^{K-1} \frac{n_j}{N} (1 - \delta_{i,j}) \\ &= \frac{1}{2} s n_{i-1}\, 2u_{i-1} \left\{ \begin{array}{ll} \left(1 - \frac{n_i}{N}\right) & i = 1 \ldots K-1 \\ 1 & i = K. \end{array} \right. \end{aligned} \tag{S40}$$

Similarly, the summation in Eq. (S39b) reduces to

$$\sigma_i^{(s)} = \tfrac{1}{2} s n_i (1 - 2u_i) \left(1 - \tfrac{n_i}{N}\right) \qquad i = 0 \ldots K-1. \tag{S41}$$

The summation in Eq. (S39c) is more involved but can be performed by partitioning its

summands into three terms as follows

$$\lambda_{j,i}^{(sm)} + \lambda_{j,i}^{(s)} = a_{i,j} + b_{i,j}\left(1-\delta_{i-1,j}\right) - b_{i,j}\left(1-\delta_{i,j}\right)$$

where

$$\begin{aligned} a_{i,j} &= \frac{1}{2} s n_j \frac{n_i}{N}\left(1-\delta_{i,j}\right) \\ b_{i,j} &= \frac{1}{2} s n_j \, 2u_j \frac{n_i}{N}. \end{aligned}$$

The summation over $a_{i,j}$ can be performed immediately

$$\sum_{j=0}^{K-1} a_{i,j} = \tfrac{1}{2} s n_i \left(1-\tfrac{n_i}{N}\right) \qquad i = 0 \dots K-1, \tag{S42}$$

since $n_K = 0$ during the course of the simulation. The summations over the terms involving $b_{i,j}$ can be written

$$\sum_{j=0}^{K-1} b_{i,j}\left(1-\delta_{i-1,j}\right) = \begin{cases} b_{0,0} + \dots + b_{0,K-1} & i = 0 \\ b_{i,0} + \dots + [b_{i,i-1}] + \dots + b_{i,K-1} & i = 1 \dots K-1 \end{cases} \tag{S43}$$

$$\sum_{j=0}^{K-1} b_{i,j}\left(1-\delta_{i,j}\right) = \begin{cases} [b_{0,0}] + \dots + b_{0,K-1} & i = 0 \\ b_{i,0} + \dots + [b_{i,i}] + \dots + b_{i,K-1} & i = 1 \dots K-1 \end{cases} \tag{S44}$$

where the square bracket indicates that the corresponding term should be omitted from the sum. Most of these terms cancel when Eq. (S44) is subtracted from Eq. (S43) yielding

$$\sum_{j=0}^{K-1} b_{i,j}\left(1-\delta_{i-1,j}\right) - \sum_{j=0}^{K-1} b_{i,j}\left(1-\delta_{i,j}\right) = \begin{cases} b_{0,0} & i = 0 \\ b_{i,i} - b_{i,i-1} & i = 1 \dots K-1 \end{cases} \tag{S45}$$

Adding Eqs. (S42) and (S45) yields

$$\Sigma_i = \begin{cases} \frac{1}{2} s n_0 \left(1-\frac{n_0}{N}\right) + \frac{1}{2} s n_0 \, 2u_0 \frac{n_0}{N} & i = 0 \\ \frac{1}{2} s n_i \left(1-\frac{n_i}{N}\right) + \frac{1}{2} s n_i \, 2u_i \frac{n_i}{N} - \frac{1}{2} s n_{i-1} \, 2u_{i-1} \frac{n_i}{N} & i = 1 \dots K-1 \end{cases} \tag{S46}$$

Plugging the expressions in Eqs. (S40), (S41) and (S46) into Eq. (S38) and performing some algebra yields

$$\Lambda_{\vec{n}}^{(i)} = \begin{cases} -n_0 u_0 & i = 0 \\ n_{i-1} u_{i-1} - n_i u_i & i = 1 \dots K-1 \\ n_{K-1} u_{K-1} & i = K \end{cases}$$

Finally, plugging this expression into Eq. (S36) yields the deterministic equations for the

feedback model

$$d\langle N_0\rangle/dt = -\langle N_0\rangle u_0 \tag{S47a}$$

$$d\langle N_i\rangle/dt = \langle N_{i-1}\rangle u_{i-1} - \langle N_i\rangle u_i \qquad i=1,\ldots,K-1 \tag{S47b}$$

$$d\langle N_K\rangle/dt = \langle N_{K-1}\rangle u_{K-1} \tag{S47c}$$

These equations are identical to those derived in the branching model at long times, Eq. (S5), as they should be.

2.3 Formal analysis of the accumulation of two mutations

Previous studies in the field of population genetics have identified a number of parameter regimes in which mutation accumulation in populations subject to genetic drift can be described using simple analytical formulae [6, 11–15]. Here, we build on this theoretical work to better understand why symmetric stem cell divisions protect against mutation accumulation and to derive mathematical formulae that accurately describe the extent of protection.

2.3.1 Derivation of PF formula

The generation of the first double-mutant stem cell in the purely asymmetric trajectory of Fig. **S2**C can be described by a pair of independent exponential random variables, X_0 and X_1, with rate parameters Nu_0 and u_1, respectively. These values for the rates follow from the fact that a population containing n_i stage-i stem cells generates a stage-$(i+1)$ stem cell for the first time at rate

$$\lambda_i^{(am)} + \sum_{j=0}^{K-1} \lambda_{ij}^{(sm)} \;=\; n_i u_i,$$

where the rates $\lambda_i^{(am)}$ and $\lambda_{ij}^{(sm)}$ are defined in Section 2.1. The time at which the first double-mutant stem cell arises, $X_0 + X_1$, is hypoexponentially distributed [16, p253] with cumulative distribution R_2 given by

$$\begin{aligned} dR_0/dt &= -Nu_0R_0 \\ dR_1/dt &= Nu_0R_0 - u_1R_1 \\ dR_2/dt &= u_1R_1 \end{aligned}$$

and initial condition $R_0(0) = 1$, $R_1(0) = 0$ and $R_2(0) = 0$. The solution is (Fig. 3E)

$$R_2(t) = H_2(Nu_0, u_1; t), \tag{S48}$$

where the 2-parameter hypoexponential distribution is given by

$$H_2(k_0, k_1; t) = \begin{cases} \frac{k_0\left(e^{-k_1t}-1\right)-k_1\left(e^{-k_0t}-1\right)}{k_1-k_0} & k_0 \neq k_1 \\ 1-(1+k_0t)\,e^{-k_0t} & k_0 = k_1. \end{cases} \tag{S49}$$

This expression for R_2 is a good approximation to the exact value provided the mean abundance of single-mutant stem cells is small, $\langle N_1(t)\rangle \ll 1$, a regime we call the "Stochastic" regime. In the alternative "Deterministic" regime, $\langle N_1(t)\rangle \gg 1$, the single-mutant abundance is well approximated by its mean, $N_1(t) \approx \langle N_1(t)\rangle$, and the cumulative risk of generating a double-mutant stem cell is

$$R_2 \;=\; 1 - e^{-u_1 \int_0^t \langle N_1(t')\rangle dt'}, \tag{S50}$$

which is a special case of Eq. (S12). To progress further analytically, we solve Eq. (S47) to get an expression for the mean single-mutant abundance

$$\langle N_1(t)\rangle = \begin{cases} \frac{Nu_0}{u_1-u_0}\left(e^{-u_0t} - e^{-u_1t}\right) & u_0 \neq u_1 \\ Nu_0te^{-u_0t} & u_0 = u_1, \end{cases} \tag{S51}$$

which can be approximated by

$$\langle N_1(t)\rangle \approx \left\{ \begin{array}{ll} Nu_0t & t \ll 1/u_1 \\ \frac{Nu_0}{u_1} & 1/u_1 \ll t \ll 1/u_0 \\ \frac{Nu_0}{u_1}e^{-u_0t} & 1/u_0 \ll t \end{array} \right\} \tag{S52}$$

provided there is a separation of timescales, $u_0 \ll u_1$. We needn't consider further the regime $t \gg 1/u_0$ because we carried out our simulations at $u_0 = 10^{-6}$ whereas biologically relevant timescales are no larger than $\sim 10^4$ cell cycles. The boundary between the Stochastic and Deterministic regimes, $\langle N_1(t)\rangle \sim 1$, is therefore defined by (Fig. 3I)

$$t \sim 1/Nu_0 \qquad\qquad t \ll 1/u_1 \tag{S53a}$$

$$N \sim u_1/u_0 \qquad\qquad 1/u_1 \ll t \ll 1/u_0. \tag{S53b}$$

Plugging Eq. (S52) into Eq. (S50), one may show that the cumulative risk in the Deterministic regime, $N \gg u_1/u_0,\ t \gg 1/Nu_0$, is

$$R_A = 1 - e^{-\frac{1}{2}Nu_0u_1t^2}. \tag{S54}$$

Let us now consider the purely symmetric trajectory in Fig. **S2**D. Many single-mutant lineages are expelled from the tissue before one arises that survives neutral drift long enough to acquire a second mutation. This occurs with probability $Q_1^{(\infty)} = \sqrt{2u_1}$ (see Eq. (S24)), and when it does, the lineage drifts for a time $\tau_1 = 2\ln 2/\sqrt{2u_1}$ before mutating (see Eq. (S30); inset of Fig. **S2**D). The single-mutant lineage destined to mutate typically does not fix in the population — an adaptation mechanism known as "Stochastic Tunneling". In this regime, the purely symmetric cumulative risk is (Fig. 3E)

$$R_S = 1 - e^{-Nu_0Q_1^{(\infty)}t} \tag{S55}$$

since the drift time is negligible compared to the mean time until the first successful single-mutant lineage, $\tau_1 \ll 1/Nu_0Q_1^{(\infty)}$ (Fig. **S2**D).

Protection vanishes at still longer time scales, $t \gg 1/Nu_0Q_1^{(\infty)}$, where both the asymmetric and symmetric cumulative risks have saturated at unity (Fig. 3E). Symmetry also

fails to protect when the tissue age is much smaller than the drift time, $t \ll \tau_1$ (Fig. 3E). Here, the second mutation typically occurs in the first single-mutant lineage (Fig. **S2**E, F) because, for this particular parameter set, single-mutants rarely arise so early,

$$P[\,T_1 < t\,] = 1 - e^{-Nu_0 t} \approx Nu_0 t \ll 1.$$

Thus T_1, the time at which the first single-mutant stem cell arose, has probability density function

$$P[\,T_1 \in (t', t' + dt')\,] \approx Nu_0 dt'.$$

Moreover Eq. (S29) tells us that, if the single-mutant lineage is founded at time t', then it yields a double mutant by time t with probability

$$P[\,T_2 < t \,|\, T_1 \in (t', t' + dt')\,] = u_1(t - t'),$$

where T_2 is the time at which the first double-mutant stem cell arose. The net cumulative risk of acquiring the double-mutant stem cell by time t is therefore (Fig. 3E)

$$\begin{aligned} R_S(t) &= \int_0^t P[\,T_2 < t \,|\, T_1 \in (t', t' + dt')\,]\, P[\,T_1 \in (t', t' + dt')\,] \\ &= \int_0^t u_1(t - t') N u_0 dt' \\ &= \frac{1}{2} N u_0 u_1 t^2, \end{aligned} \tag{S56}$$

irrespective of the symmetry fraction. This expression coincides with the purely asymmetric risk derived in Eq. (S48) since $t \ll \min\{1/Nu_0, 1/u_1\}$ (for the parameters of Fig. **S2**C-F). In this "short-time" regime ($t \ll \tau_1$, Fig. 3I), the beneficial effect of symmetric extinctions are cancelled by the deleterious expansion of single-mutant clones (Fig. 1C, D). Fig. **S2**G–I shows that the short-time regime can extend to tissue ages as large as 10^3 stem cell cycles if the secondary mutation rate is small enough, $u_1 \ll 1/t^2$, explaining why protection vanishes in the lower part of Fig. 3D. Stochastic tunneling occurs for population sizes up to $N = 1/u_0$, beyond which the symmetric single-mutant abundance is approximately deterministic [6, 13] and protection is lost (see Section 2.3.2).

When the population size is small, the probability that a single-mutant clone fixes in the population exceeds the probability that it mutates before fixing, $1/N \gg Q_1^{(\infty)}$ (Fig. 3I). This is the "Sequential Fixation" regime [6, 13] where it becomes possible that the first double-mutant stem cell arises after fixation of the corresponding single-mutant lineage. To make analytic progress in this regime, we consider only sufficiently large times, $t \gg \tau_1$, allowing us to neglect the fixation time (which is of order $N \ll \tau_1$). The probability that a single-mutant

lineage founded at time $t = 0$ mutates by some later time t via sequential fixation is

$$
\begin{aligned}
P[\,T_2 < t\,,\,\text{fix}\,] &= P[\,T_2 < t\,|\,\text{fix}\,]\,P[\text{fix}\,] \\
&= \left(1 - e^{-Nu_1 t}\right)\frac{1}{N} \\
&= \begin{cases} u_1 t & t \ll 1/Nu_1 \\ 1/N & t \gg 1/Nu_1 \end{cases}
\end{aligned}
\tag{S57}
$$

whereas via stochastic tunneling it is

$$
P[\,T_2 < t\,,\,\text{no fix}\,] = Q_1^{(\infty)}.
$$

Using the fact that $\tau_1 \ll 1/Nu_1$, one may then show that the sequential fixation route is more likely than the stochastic tunneling route, $P[\,T_2 < t\,,\,\text{fix}\,] \gg P[\,T_2 < t\,,\,\text{no fix}\,]$.

At long times (Fig. 3I),

$$
t \gg 1/Nu_1, \tag{S58}
$$

one may neglect the time between (instantaneous) fixation of the single-mutant lineage and acquisition of the second mutation (Fig. **S2**L). Thus the cumulative symmetric risk in this regime is determined by the rate at which single mutants destined for fixation arise. This rate is u_0 (single-mutant lineages arise at rate Nu_0 and fix with probability $1/N$) implying that the net cumulative risk is (Fig. **S2**J)

$$
R_S = 1 - e^{-u_0 t}. \tag{S59}
$$

On long time scales, $t \gg 1/Nu_1$, symmetric stem cell extinctions flush many single-mutant lineages out of the tissue until one survives drift, fixes, and mutates leading to significant protection (Fig. **S2**M). At short times, $\tau_1 \ll t \ll 1/Nu_1 \leq 1/Nu_0$ (we assume that $u_1 \geq u_0$), the second mutation typically occurs in the first single-mutant lineage (Fig. **S2**O). Furthermore, Eq. (S57) implies that

$$
P[\,T_2 < t\,|\,T_1 \in (t', t' + dt')\,] = u_1(t - t').
$$

We may therefore proceed exactly as we did for the Stochastic Tunneling regime at short times obtaining Eq. (S56), which again coincides with the purely asymmetric risk derived in Eq. (S48) since $t \ll 1/Nu_1 \leq \min\{1/Nu_0, 1/u_1\}$. The beneficial effect of symmetric extinctions is thus cancelled by the deleterious effect of clonal expansion, as we found in the Stochastic Tunneling regime at short times.

In summary, symmetry is protective provided the tissue cycles rapidly and/or the *secondary* mutation rate is high (Fig. **S3**A-C shows that symmetry is not necessarily protective if instead the *primary* mutation rate is fast). Together, Eqs. (S48), (S54), (S55) and (S59) can be used to estimate PF $= R_A/R_S$ to an accuracy of 40% (Fig. **S2**P–R) throughout the protected part of parameter space (Figs. 3I–L).

2.3.2 Deterministic regime of a purely symmetric population

Fig. **S3**B shows that when the average number of new single mutants produced per cell cycle becomes large, $Nu_0 \gg 1$, the purely symmetric single-mutant abundance is well approximated by its mean. This regime is included in the Deterministic regime of a purely asymmetric population, defined by Eq. (S53) (see also Fig. 3I), and so the cumulative risks coincide (Fig. **S3**C). When $Nu_0 \gg 1$, the time at which the first successful single-mutant lineage appears is small compared to its drift time, $1/Nu_0 Q_1^{(\infty)} \ll \tau_1$. In other words, the rate of progression is limited by the time taken for a successful single-mutant lineage to drift before mutating. This assumes however that the first such lineage gives rise to the first double-mutant stem cell. The fact that the mean first passage time to the double mutant (approximately the time at which cumulative risk reaches 50% in Fig. **S3**C) is significantly smaller than τ_1 indicates instead that many single-mutant lineages founded early on compete against one another until one acquires the first double-mutant stem cell improbably early. We conclude that, in the Deterministic regime of a symmetric population, protective clonal extinctions are out-striped by the rapid production of single-mutant stem cells.

3 Unordered accumulation of two mutations

In this section we generalize the models of mutation accumulation presented in Sections 1 and 2 to include the possibility that loci mutate in any order. We consider only the case of two loci, A and B.

3.1 "Branching" model

Assume stem cell divisions are synchronized. A wild-type cell, X_0 (where X is either a stem cell S or non-stem cell D), acquires a mutation at locus A with probability $u_{0\to aB}$ and at locus B with probability $u_{0\to Ab}$ per stem-cell division, independent of the fate of its sister cell. Each of these single mutants, $X_{i\in\{aB,Ab\}}$, becomes a double-mutant, X_2, with probability $u_{i\to 2}$ per stem cell division. All other transitions (e.g. $0 \to 2$) are not permitted in a single stem-cell division ($u_{0\to 2} = 0$). Following Section 1.1, one may show that the division of a wild-type stem cell results in one of seven possible outcomes with the following probabilities:

$$S_0 \longrightarrow \left\{ \begin{array}{lrcl} S_0 + S_0 & p_{0,r} & = & \frac{1}{2}s\,[1 - 2(u_{0\to aB} + u_{0\to Ab})] \\ S_0 + S_{aB} & p_{0,0\xrightarrow{r}aB} & = & \frac{1}{2}s\,2u_{0\to aB} \\ S_0 + S_{Ab} & p_{0,0\xrightarrow{r}Ab} & = & \frac{1}{2}s\,2u_{0\to Ab} \\ S_0 & p_{0,a} & = & (1-s)\,[1 - (u_{0\to aB} + u_{0\to Ab})] \\ S_{aB} & p_{0,0\xrightarrow{a}aB} & = & (1-s)u_{0\to aB} \\ S_{Ab} & p_{0,0\xrightarrow{a}Ab} & = & (1-s)u_{0\to Ab} \\ \emptyset & p_{0,e} & = & \frac{1}{2}s \end{array} \right\} \quad \text{(S60)}$$

Similarly, the division of a single-mutant stem cell with genotype $i \in \{aB, Ab\}$ results in one of five possible outcomes:

$$S_i \longrightarrow \left\{ \begin{array}{llcl} S_i + S_i & p_{i,r} & = & \frac{1}{2}s\,(1-2u_{i\to 2}) \\ S_i + S_2 & p_{i,i \xrightarrow{r} 2} & = & \frac{1}{2}s\,2u_{i\to 2} \\ S_i & p_{i,a} & = & (1-s)(1-u_{i\to 2}) \\ S_2 & p_{i,i \xrightarrow{a} 2} & = & (1-s)u_{i\to 2} \\ \emptyset & p_{i,e} & = & \frac{1}{2}s \end{array} \right\} \tag{S61}$$

The update rules for the random population sizes, N_i, of each of the four possible genotypes are

$$\begin{aligned} N_0(t+1) &= N_0(t) - B_0(t) \\ N_i(t+1) &= N_i(t) + A_{0\to i}(t) - B_i(t) \qquad\qquad i \in \{aB, Ab\} \\ N_2(t+1) &= N_2(t) + A_{aB\to 2}(t) + A_{Ab\to 2}(t) \end{aligned}$$

where

$$\begin{aligned} A_{0\to i}(t) &= Z_{0,0 \xrightarrow{r} i}(t) + Z_{0,0 \xrightarrow{a} i}(t) \qquad\qquad i \in \{aB, Ab\} \\ A_{i\to 2}(t) &= Z_{i,i \xrightarrow{r} 2}(t) + Z_{i,i \xrightarrow{a} 2}(t) \qquad\qquad i \in \{aB, Ab\} \\ B_0(t) &= Z_{0,0 \xrightarrow{a} aB}(t) + Z_{0,0 \xrightarrow{a} Ab}(t) + Z_{0,e}(t) - Z_{0,r}(t) \\ B_i(t) &= Z_{i,i \xrightarrow{a} 2}(t) + Z_{i,e}(t) - Z_{i,r}(t) \qquad\qquad i \in \{aB, Ab\} \end{aligned}$$

and $\vec{Z}_{i\in\{0,aB,Ab\}}$ are drawn from the multinomial distribution, Eq. (S3).

3.2 "Moran" model

We formulate the model analogously to Section 2.1. The net rate of asymmetric divisions that decrement the number of stem cells with genotype $i \in \{0, aB, Ab\}$, n_i, while incrementing the number of stem cells with genotype $k \in \{aB, Ab, 2\}$ is

$$\lambda_{ik}^{(am)} = (1-s)n_i u_{i\to k}\,,$$

where s is the probability that a stem cell division is symmetric. There are two types of replacements. In the first type, which occurs at rate

$$\lambda_{ijk}^{(sm)} = \tfrac{1}{2}s n_i 2u_{i\to k}\tfrac{n_j}{N}\,(1-\delta_{jk})\ ,$$

a stem cell with genotype $i \in \{0, aB, Ab\}$ is chosen for symmetric renewal, a stem cell with genotype $j \in \{0, aB, Ab\}$ is chosen for symmetric extinction, and a stem cell with genotype $k \in \{aB, Ab, 2\}$ is produced by mutation of one of the daughters of the symmetric renewal. This type of replacement thus increments the k-population and decrements the j-population

such that the total number of stem cells, N, remains unchanged. Alternatively, with rate

$$\lambda_{ij}^{(s)} = \tfrac{1}{2} s n_i \left(1 - \textstyle\sum_k 2u_{i\to k}\right) \tfrac{n_j}{N} \left(1 - \delta_{i,j}\right) \qquad i, j \in \{0, aB, Ab\},$$

the symmetric renewal is of the form $S_i \to S_i + S_i$, in which case the i-population is incremented whereas the j-population is decremented.

3.3 Deterministic solution

Following Section 1.3, one may show that the average population sizes obey

$$\begin{aligned} d\langle N_0\rangle/dt &= -\langle N_0\rangle (u_{0\to aB} + u_{0\to Ab}) \\ d\langle N_i\rangle/dt &= \langle N_0\rangle u_{0\to i} - \langle N_i\rangle u_{i\to 2} \qquad\qquad i \in \{aB, Ab\} \\ d\langle N_2\rangle/dt &= \langle N_{aB}\rangle u_{aB\to 2} + \langle N_{Ab}\rangle u_{Ab\to 2} \end{aligned}$$

The solution of these equations is

$$\langle N_0\rangle = N e^{-(u_{0\to aB} + u_{0\to Ab})t} \tag{S65a}$$

$$\langle N_i\rangle = \frac{N u_{0\to i}}{u_{i\to 2} - (u_{0\to aB} + u_{0\to Ab})} \left(e^{-(u_{0\to aB}+u_{0\to Ab})t} - e^{-u_{i\to 2}t}\right) \qquad i \in \{aB, Ab\} \tag{S65b}$$

$$\langle N_2\rangle = \sum_{i\in\{aB,Ab\}} \frac{N u_{0\to i}}{u_{0\to aB} + u_{0\to Ab}} H_2(u_{0\to aB} + u_{0\to Ab}, u_{i\to 2}; t) \tag{S65c}$$

where H_2 is the 2-parameter hypoexponential distribution defined in Eq. (S49).

3.4 Purely asymmetric risk

Eq. (S11) tells us that the probability that the first double-mutant stem cell arises by time t is

$$R_2(t) = 1 - \left(1 - \frac{\langle N_2(t)\rangle}{N}\right)^N. \tag{S66}$$

3.5 Purely symmetric risk when loci mutate independently

Consider the case depicted in Fig. 5B where $t \gg \tau_{aB} = \frac{2\ln 2}{\sqrt{2u_{aB\to 2}s}}$. Some double mutants arise from aB single mutants via stochastic tunneling whereas others arise from a deterministic background of Ab single mutants. The net rate of mutation accumulation is approximately the sum of two independent Poisson processes with rates

$$k_{aB} = N u_{0\to aB} Q_{aB}^{(\infty)},$$

where $Q_{aB}^{(\infty)} = \sqrt{2u_{aB\to 2}}$, and

$$k_{Ab}(t) = \langle N_{Ab}(t)\rangle u_{Ab\to 2},$$

respectively [6]. For the parameter values of Fig. 5B,

$$\langle N_{Ab}(t)\rangle \approx N(1 - e^{-u_{0\to Ab}t}), \tag{S67}$$

and the cumulative risk of generating a double-mutant stem cell is

$$\begin{aligned} P[T_2 < t] &\approx 1 - \exp\left(-\int_0^t [k_{aB} + k_{Ab}(t')]\, dt'\right) \\ &\approx 1 - \exp\left(-k_{aB}t - Nu_{Ab\to 2}\left(t - \frac{1 - e^{-u_{0\to Ab}t}}{u_{0\to Ab}}\right)\right) \end{aligned} \tag{S68}$$

To combine mutational paths at short times, $t \ll \tau_{aB}$, it is helpful to introduce a random variable $Y_1 \in \{S_{aB}, S_{Ab}\}$ representing the genotype of the single-mutant stem cell that generates the first double-mutant stem cell. In cases where an aB stem cell generates the first double mutant ($Y_1 = S_{aB}$), typically it is the first aB stem cell that does so (Fig. **S3**E). Therefore, we may invoke the argument leading to Eq. (S56) obtaining

$$P[\,T_2 < t, Y_1 = S_{aB}\,] = \frac{1}{2} N u_{0\to aB} u_{aB\to 2} t^2. \tag{S69}$$

In the alternate scenario — the double mutant arises from a deterministic background of Ab single mutants — the cumulative risk is

$$\begin{aligned} P[\,T_2 < t, Y_1 = S_{Ab}\,] &\approx 1 - \exp\left(-\int_0^t k_{Ab}(t')\, dt'\right), \\ &\approx \frac{1}{2} N u_{0\to Ab} u_{Ab\to 2} t^2 \end{aligned} \tag{S70}$$

for the parameter values of Fig. **S3**D-H. The mutational paths are combined by simply adding the cumulative risks in Eqs. (S69) and (S70) obtaining (Fig. **S3**H)

$$P[\,T_2 < t\,] = \frac{1}{2} N u_{0\to aB} u_{aB\to 2} t^2 + \frac{1}{2} N u_{0\to Ab} u_{Ab\to 2} t^2. \tag{S71}$$

4 Effect of selection on ordered mutation accumulation

In our analysis thus far, we assumed each stem cell contributes on average one stem cell to the population one cell cycle later. This is reasonable for asymmetric stem cell divisions but for symmetric divisions, renewals and symmetric extinctions could be imbalanced (ie. subject to selection). Here, we relax the assumption of neutrality by allowing the mean number of stem cell descendants contributed by a given parent to depend on its stage, subject to the constraint that the net stem cell population size is conserved.

4.1 Formulation

Leaving the asymmetric divisions in the Moran model of Section 2.1 unchanged (i.e. retaining the rates in Eq. (S31)), we generalize the model such that, when a stem cell replacement

occurs, a stage-i stem cell is chosen for symmetric renewal with probability $\frac{w_i}{\sum_k w_k n_k}$, where w_i is the fitness of a stage-i stem cell and n_i is the number of such stem cells. Thus, the rates of replacements become

$$\lambda_{ij}^{(sm)} = \frac{1}{2} s \frac{w_i}{\overline{w}} n_i 2u_i \frac{n_j}{N}(1-\delta_{i+1,j}) \tag{S72a}$$

$$\lambda_{ij}^{(s)} = \frac{1}{2} s \frac{w_i}{\overline{w}} n_i (1-2u_i) \frac{n_j}{N}(1-\delta_{i,j}) \tag{S72b}$$

where $i, j = 0 \dots K-1$ index the stages, s is the fraction of stem cell divisions that are symmetric, and

$$\overline{w} = \sum_k w_k \frac{n_k}{N}$$

is the mean fitness of the population. Notice that when all stages have equal fitness the transition rates in Eq. (S72) reduce to Eqs. (S32) and (S33) for a neutral stem cell population.

4.2 Deterministic Analysis

In this section, we generalize the deterministic equations, Eq. (S47), to incorporate selection. As shown in Section 2.2, the mean number of stage-i stem cells, $\langle N_i \rangle$, evolves according to

$$d\langle N_i(t)\rangle/dt = \sum_{\vec{n}} \Lambda_{\vec{n}}^{(i)} P(\vec{n}, t), \tag{S73}$$

where $\vec{n} = (n_0 \dots n_K)$ represents the numbers of stage-$0 \dots K$ stem cells in a population and $P(\vec{n}, t)$ is the probability of finding the population in the configuration $\vec{n}$ at time t. Repeating the derivation of $\Lambda_{\vec{n}}^{(i)}$ presented in Section 2.2, this time using the replacement rates defined by Eq. (S72), we obtain

$$\Lambda_{\vec{n}}^{(i)} = \begin{cases} -\left[(1-s) + s\frac{w_0}{\overline{w}}\right] n_0 u_0 + \frac{1}{2}s\left(\frac{w_0}{\overline{w}} - 1\right) n_0 & i = 0 \\ \left[(1-s) + s\frac{w_{i-1}}{\overline{w}}\right] n_{i-1} u_{i-1} - \left[(1-s) + s\frac{w_i}{\overline{w}}\right] n_i u_i + \frac{1}{2}s\left(\frac{w_i}{\overline{w}} - 1\right) n_i & i = 1 \dots K-1 \\ \left[(1-s) + s\frac{w_{K-1}}{\overline{w}}\right] n_{K-1} u_{K-1} & i = K \end{cases}$$

In contrast to the neutral case, Eqs. (S73) are no longer closed and depend upon factors of the form $\langle N_i/\overline{w}\rangle$, for which one must derive further differential equations. We can truncate this hierarchy by making the approximation $\vec{N} \approx \langle \vec{N} \rangle$ leading to

$$d\langle N_0\rangle/dt \approx -f_0 \langle N_0\rangle u_0 + \frac{1}{2}s\left(\frac{w_0}{\langle\overline{w}\rangle} - 1\right)\langle N_0\rangle \tag{S74a}$$

$$d\langle N_i\rangle/dt \approx f_{i-1}\langle N_{i-1}\rangle u_{i-1} - f_i \langle N_i\rangle u_i + \frac{1}{2}s\left(\frac{w_i}{\langle\overline{w}\rangle} - 1\right)\langle N_i\rangle \quad i = 1, \dots, K-1 \tag{S74b}$$

$$d\langle N_K\rangle/dt \approx f_{K-1}\langle N_{K-1}\rangle u_{K-1} \tag{S74c}$$

where the "ensemble average" of the mean population fitness is

$$\langle \overline{w}(t) \rangle = \sum_k w_k \frac{\langle N_k(t) \rangle}{N}$$

and

$$f_i(t) = (1-s) + s\frac{w_i}{\langle \overline{w}(t) \rangle}.$$

This "mean-field approximation" is expected to fail whenever one or more stages are small, under which circumstances the distribution $P(\vec{n}, t)$ becomes very broad and is poorly approximated by its mean.

5 Protection in the compartmentalized intestine

In an intestine undergoing a purely asymmetric pattern of division, each stem cell behaves independently of all the others. Thus, as argued in Section 1.4, the intestine-wide risk of ordered accumulation of K mutations with mutation rates $u_0, \ldots, u_{K-1}$ is (red lines in Fig. **S7**)

$$R_A = 1 - [1 - H_K(u_0, \ldots, u_{K-2}, u_{K-1}; t)]^{NM}, \tag{S75}$$

where N is the number of stem cells per crypt and M is the number of crypts. The function $H_K(k_0, \ldots, k_{K-1}; t)$ is the K-parameter hypoexponential distribution [16, p253], which we computed by solving the ordinary differential equations

$$\begin{aligned} dP_0/dt &= -P_0 k_0 \\ dP_i/dt &= P_{i-1}k_{i-1} - P_i k_i \qquad i = 1, \ldots, K-1 \\ dH_K/dt &= P_{K-1}k_{K-1} \end{aligned}$$

with initial conditions $P_0 = 1, P_i = 0\,(i = 1, \ldots, K-1), H_K = 0$. In the purely symmetric case (under the Moran model; Section 2.1), the K-fold mutant arises in an individual crypt via sequential fixations (provided the number of stem cells per crypt and the mutation rates are small enough [6, 17]) implying that the intestine-wide risk is (green lines in Fig. **S7**)

$$R_S = 1 - [1 - H_K(u_0, \ldots, u_{K-2}, N u_{K-1}; t)]^{M}. \tag{S76}$$

6 Effect of allowing for simultaneous mutations in both daughter stem cells

In Section 1.1, we formulated the "Branching" model of ordered mutation accumulation by neglecting the possibility that, upon division of a stem cell, both daughter cells may simultaneously mutate. To find out whether this approximation is good, we re-formulate the model to include this possibility. Retaining probability contributions of order u_i^2, one finds that the division of a stem cell S_i $(i = 0 \ldots K-1)$ now results in one of six possible

outcomes with the following probabilities:

$$S_i \longrightarrow \left\{ \begin{array}{lrcl} S_i + S_i & p_{i,r} & = & \frac{1}{2}s(1-2u_i+u_i^2) \\ S_i + S_{i+1} & p_{i,rm} & = & \frac{1}{2}s\,2u_i(1-u_i) \\ S_{i+1} + S_{i+1} & p_{i,rm2} & = & \frac{1}{2}s\,u_i^2 \\ S_i & p_{i,a} & = & (1-s)(1-u_i) \\ S_{i+1} & p_{i,am} & = & (1-s)u_i \\ \emptyset & p_{i,e} & = & \frac{1}{2}s \end{array} \right\} \tag{S77}$$

Let $Z_{ij}(t)$ be the (random) number of times that a category-j division in Eq. (S77) occurs at time t in a population of stage-i stem cells of (random) size $N_i(t)$. The update rules for the N_i are again given by Eq. (S2), where this time

$$\begin{aligned} A_i(t) &= 2Z_{i,rm2}(t) + Z_{i,rm}(t) + Z_{i,am}(t) \\ B_i(t) &= Z_{i,rm2}(t) + Z_{i,am}(t) + Z_{i,e}(t) - Z_{i,r}(t), \end{aligned}$$

but $\vec{Z}_i$ is again sampled from the multinomial distribution in Eq. (S3). The recursive equations for the average population sizes again follow Eq. (S4). We re-ran stochastic simulations using the more exact algorithm formulated here; the results are unchanged (compare Fig. **S8** with Fig. 1G).

7 Supporting References

[1] T E Harris. *The Theory of Branching Processes.* Dover, 1963.

[2] Charles J Mode. *Multitype branching processes : theory and applications.* American Elsevier Pub. Co., New York, 1971.

[3] Ivana Bozic, Tibor Antal, Hisashi Ohtsuki, Hannah Carter, Dewey Kim, Sining Chen, Rachel Karchin, Kenneth W Kinzler, Bert Vogelstein, and Martin A Nowak. Accumulation of driver and passenger mutations during tumor progression. *Proceedings of the National Academy of Sciences*, 107(43):18545–18550, 2010.

[4] Norman T J Bailey. *The elements of stochastic processes, with applications to the natural sciences.* Wiley, New York, 1964.

[5] L de Haan and Ana Ferreira. *Extreme value theory: an introduction.* Springer, 2006. URL `http://dx.doi.org/10.1007/0-387-34471-3`.

[6] Daniel B Weissman, Michael M Desai, Daniel S Fisher, and Marcus W Feldman. The rate at which asexual populations cross fitness valleys. *Theoretical Population Biology*, 75(4):286–300, 2009.

[7] Samuel Karlin and Howard M Taylor. *A first course in stochastic processes.* Academic Press, New York, 2nd edition, 1975.

[8] Marek Kimmel and David E Axelrod. *Branching processes in biology*. Springer, New York, 2002.

[9] P A P Moran. Random processes in genetics. *Mathematical Proceedings of the Cambridge Philosophical Society*, 54(01):60, 1958.

[10] P A P Moran. *The Statistical processes of evolutionary theory*. Clarendon, Oxford, 1962.

[11] Natalia L Komarova, Anirvan Sengupta, and Martin A Nowak. Mutation–selection networks of cancer initiation: tumor suppressor genes and chromosomal instability. *Journal of Theoretical Biology*, 223(4):433–450, 2003.

[12] Yoh Iwasa, Franziska Michor, and Martin A Nowak. Stochastic tunnels in evolutionary dynamics. *Genetics*, 166(3):1571–1579, 2004.

[13] Martin A Nowak, Franziska Michor, Natalia L Komarova, and Yoh Iwasa. Evolutionary dynamics of tumor suppressor gene inactivation. *Proceedings of the National Academy of Sciences of the United States of America*, 101(29):10635–8, 2004.

[14] Yoh Iwasa, Franziska Michor, Natalia L Komarova, and Martin A Nowak. Population genetics of tumor suppressor genes. *Journal of Theoretical Biology*, 233(1):15–23, 2005.

[15] Rick Durrett, Deena Schmidt, and Jason Schweinsberg. A waiting time problem arising from the study of multi-stage carcinogenesis. *The Annals of Applied Probability*, 19(2): 676–718, 2009.

[16] Sheldon M Ross. *Introduction to probability models*. Academic Press, New York, 7th edition, 2000.

[17] Martin A Nowak, Natalia L Komarova, Anirvan Sengupta, Prasad V Jallepalli, Ie-Ming Shih, Bert Vogelstein, and Christoph Lengauer. The role of chromosomal instability in tumor initiation. *Proceedings of the National Academy of Sciences*, 99(25):16226–16231, January 2002.

[18] Chris Hornsby, Karen M Page, and Ian Tomlinson. The in Vivo Rate of Somatic Adenomatous Polyposis Coli Mutation. *The American Journal of Pathology*, 172(4): 1062–1068, April 2008.

[19] Bruce M Boman and Emina Huang. Human Colon Cancer Stem Cells: A New Paradigm in Gastrointestinal Oncology. *Journal of Clinical Oncology*, 26(17):2828–2838, January 2008.

[20] Satoshi Nishimura, Naoki Wakabayashi, Kazuyuki Toyoda, Kei Kashima, and Shoji Mitsufuji. Expression of Musashi-1 in Human Normal Colon Crypt Cells: A Possible Stem Cell Marker of Human Colon Epithelium. *Digestive Diseases and Sciences*, 48(8): 1523–1529, 2003.

[21] Pierre Nicolas, Kyoung-Mee Kim, Darryl Shibata, and Simon Tavaré. The Stem Cell Population of the Human Colon Crypt: Analysis via Methylation Patterns. *PLoS Computational Biology*, 3(3):e28, March 2007.

8 Supporting Figure Legends

Figure S1: Numerical Screen
(A) Concordance of "Moran" and "Branching" models used to screen large and small populations, respectively. The lifetime cumulative risk of accumulating two mutations in a symmetric population was computed for a variety of stem-cell population sizes and organism lifetimes under both models (right panels). The models predict the same cumulative risk over most of parameter space but differ significantly in small populations at large lifetimes where extinctions of the entire stem cell population in the Branching model reduce risk by at least a factor of two (white contour in left panel). (B) Parameter sets comprising the numerical screen of Table S1 were classified into 4 types based on the number of stochastic stages. Representative symmetric trajectories are shown. Notice the correlation between the number of stochastic stages and mean PF (averaged over all parameter sets with a given number of stochastic stages).

Figure S2: Analysis of Stochastic Tunneling and Sequential Fixation regimes (A, B) A single wild-type stem cell was simulated until either one of its descendants mutated (with probability $Q_0^{(\infty)}$) or its lineage extinguished without mutating (with probability $1 - Q_0^{(\infty)}$). The mean time that a branching lineage drifts before mutating, τ_0, was recorded in those cases where mutation occurred. Panel A shows that the simulated lineage mutation probability (symbols) is well described by Eq. (S24) (lines) whereas panel B shows that the simulated drift time (symbols) is well described by Eq. (S30) (lines). (C-F) Typical dynamics at long times (C, D) and short times (E, F) prior to the production of the first double-mutant stem cell (yellow lightning bolt). Inset to (D) is a magnified view of the last few generations of the simulated dynamics. Population size is $N = 10^3$ stem cells and mutation rates are $u_0 = 10^{-6}$ and $u_1 = 10^{-3}$. (G – I) Protection vanishes for small secondary mutation rate, $u_1 \ll 1/L^2$. In these panels, population size is $N = 10^3$ stem cells and mutation rates are $u_0 = 10^{-6}$ and $u_1 = 10^{-8}$. (G) Simulated (symbols) and theoretical (line, Eq. (S56)) cumulative risk. (H, I) Typical trajectories that generate a double-mutant stem cell by end of life, $T_2 < L = 10^3$cc. In both cases, one of the first few single-mutant lineages to arise from the wild-type background produces a double-mutant stem cell that arises improbably early in its parent single-mutant lineage, $T_2 \ll \tau_1 \ll 1/u_1$. (J – O) Sequential Fixation Regime. In these panels, population size is $N = 10$ stem cells and mutation rates are $u_0 = 10^{-6}$, $u_1 = 10^{-4}$. (J) Cumulative risk calculated using simulation (symbols) and Eqs. (S48), (S56) and (S59) (lines). (K, N) A double-mutant stem cell typically arises in the first single-mutant stem cell in a purely asymmetric population. (L, O) Dynamics in a purely symmetric population. (L) At long times, $t \gg 1/Nu_1$, single-mutant lineages frequently extinguish before one survives drift, fixes in the population, and then rapidly acquires the next mutation. The time taken to fix, N, and to acquire the second mutation once fixed, $1/Nu_1$, are negligible compared to the time taken for a single-mutant lineage destined for fixation to arise, $1/u_0$. (O) At short times, $t \ll 1/Nu_1$, the time between fixation and mutation cannot be neglected. (M) Symmetric extinctions outcompete fixation events to reduce mutation accumulation risk. (P – R) Accuracy of piecewise analytic formula for PF measured by the ratio of analytically computed PF (Fig. 3J–L) to exact PF as calculated via Monte Carlo simulation (Fig. 3F-H). Grey contours delineate regions (red) where the fractional error of the analytic formulae $|\mathrm{PF}_{analytic} - \mathrm{PF}_{exact}| / \mathrm{PF}_{exact}$ is less than 40%, including practically all the protected zone ($\mathrm{PF}_{exact} > 2$; white contour). Panels A and B were generated under the Branching model, Eq. (S1), whereas panels C – R were generated using the Moran model, Section 2.1. In panels C – O, purely asymmetric (symmetric) trajectories are in red (blue). cc, cell cycles.

Figure S3: Unordered "fast" and "slow" mutations
(A – C) A "fast-slow" ordered pathway. (A, B) The abundance of Ab stem cells is approximated by its mean value, $\langle N_1(t)\rangle \approx N(1-e^{-u_0 t})$, which follows from Eq. (S51) when t, $1/u_0 \ll 1/u_1$. (C) Simulated cumulative risk (symbols) is approximated by $1-\exp\left(-\int_0^t \langle N_1(t')\rangle\, u_1\, dt'\right)$. (D – H) Dynamics at short times, $t \ll \tau_{aB}$, of unordered "fast" and "slow" loci. The black line in panels F and G is Eq. (S67). (H) Simulated cumulative risk (symbols) is approximated by Eq. (S71). In all panels, population size is $N = 10^4$ stem cells and mutation rates are 10^{-2} ("fast") and 10^{-6} ("slow"). Time courses are plotted until the first double-mutant stem cell appears in the entire stem cell population.

Figure S4: Clonal extinctions out-compete progression in the intestine
(A) Model of mutation accumulation in the intestine. (B) Typical dynamics showing how various patterns of division generate the triple-mutant stem cell in the un-compartmentalized case. (C) Dynamics in a single crypt of a compartmentalized intestine. The purely asymmetric trajectory ($s = 0\%$) is representative of all trajectories examined whereas the mixed (s=10%) and purely symmetric (s=100%) trajectories show the most frequently observed type since all four possible combinations of stochastic tunneling and sequential fixation were observed at appreciable frequencies (see also [17]). The intestine was assumed to comprise 10^6 crypts, each containing 10 stem cells. Mutation rates are 10^{-6} (slow) and 10^{-3} (fast).

Figure S5: Protection persists when selection acts on stochastic stages
Protection against ordered accumulation of $K = 2$ mutations after 1000 stem cell cycles for symmetry fractions $s = 100\%$ (A) and 10% (B), calculated by Monte Carlo simulation of the generalized model presented in Section 4.1. The selection coefficient is defined in the model by $(w_1 - w_0)/w_0$, where w_i is the fitness of stage i (see Section 4.1). The insensitivity of PF to wide variations in the selection coefficient is an example of the general principle in population genetics that selection is ineffective provided the magnitude of the selection coefficient is smaller than the inverse population size. Mutation rates are $u_0 = 10^{-6}$ and $u_1 = 10^{-3}$ per locus per stem cell cycle.

Figure S6: "Increasing" mutation rates yield a broad distribution of latencies
Probability distributions of times at which the first double-mutant stem cell arose in a population of $N = 10^4$ symmetrically dividing stem cells, from simulations of the discrete-time branching process defined by Eq. (S1) (bars) and from the probability mass function $P[T_2 = t] = k(t)\exp\left(-\int_0^t k(t')\,dt'\right)$ (lines). In the Deterministic regime (A), the rate constant is given by $k = \langle N_1\rangle\, u_1$, where the mean abundance of single-mutant stem cells is $\langle N_1\rangle \approx N(1-e^{-u_0 t})$, whereas it is $k = N u_0 \sqrt{2u_1}$ in the Stochastic Tunneling regime (B). When the mutation rates are decreasing, the distribution of latency until the first double-mutant stem cell is narrow (A), but when the mutation rates are increasing, the distribution becomes wider (B), even at the same mean latency (858 cell cycles in both cases). Histogram bar height represents the probability that the mutation occurred between the bar edges. Insets show typical stochastic realizations (red) and mean single-mutant abundance (black).

Figure S7: Protection in the human colon

Cumulative risk of ordered accumulation of $K = 4$ mutations by ages 30 (A, C) and 60 (B, D), assuming mutation rates increase 1000-fold (A, B) or 100-fold (C, D) during the course of mutation accumulation from an initial rate of $u_0 = 5 \times 10^{-7}$ per locus per stem cell cycle [18]. Lines are Eqs. (S75) and (S76) whereas symbols are Monte Carlo simulations (under the Moran model; Section 2.1). The colon is assumed to be compartmentalized into $M = 10^7$ crypts, ($\sim 10^4$ crypts/cm^2 $\times$ $\sim 10^3$cm^2/colon [19]) each containing $N = 20$ stem cells [20, 21] dividing purely asymmetrically (red) or symmetrically (green) 100 times per year. Mutation rates of consecutive stages (u_0, u_1, u_2, u_3) are (A) $5 \times 10^{-7}, 5 \times 10^{-7}, 5 \times 10^{-6}, 5 \times 10^{-4}$; (B) $5 \times 10^{-7}, 5 \times 10^{-7}, 5 \times 10^{-7}, 5 \times 10^{-4}$; (C) $5 \times 10^{-7}, 5 \times 10^{-7}, 5 \times 10^{-5}, 5 \times 10^{-5}$; (D) $5 \times 10^{-7}, 5 \times 10^{-7}, 5 \times 10^{-6}, 5 \times 10^{-5}$.

Figure S8: Symmetry protects even when mutations may occur simultaneously in both daughter stem cells

The more accurate ordered mutation accumulation model presented in Section 6 was used to generate a distribution of PFs over a random ensemble of parameter sets equivalent to that used in Fig. 1G (Materials and Methods; Table S1). The distribution is unchanged (within sampling error).
